# Enhancing agricultural commodity price forecasting with deep learning

**DOI:** 10.1038/s41598-025-05103-z

**Published:** 2025-07-01

**Authors:** R. L. Manogna, Vijay Dharmaji, S. Sarang

**Affiliations:** Department of Economics and Finance, Birla Institute of Technology and Science, Pilani, K K Birla Goa Campus, Zuari nagar, Sancoale, 403726 Goa India

**Keywords:** Time-series modeling, Deep learning, Machine learning, Price forecasting, Agri commodities, Environmental economics, Energy economics

## Abstract

Accurate forecasting of agricultural commodity prices is essential for market planning and policy formulation, especially in agriculture-dependent economies like India. Price volatility, driven by factors such as weather variability and market demand fluctuations, poses significant forecasting challenges. This study evaluates the performance of traditional stochastic models, machine learning techniques, and deep learning approaches in forecasting the prices of 23 commodities using daily wholesale price data from January 2010 to June 2024. Models assessed include Autoregressive Integrated Moving Average, Support Vector Regression, Extreme Gradient Boosting, Multilayer Perceptron, Recurrent Neural Networks, Long Short-Term Memory Networks, Gated Recurrent Units, and Echo State Networks. Results show that deep learning models, particularly Long Short-Term Memory and Gated Recurrent Units, outperform others in capturing complex temporal patterns, achieving superior accuracy across error metrics. The results indicate that deep learning models, particularly Long Short-Term Memory (LSTM) networks and Gated Recurrent Units (GRU), demonstrate superior performance in capturing complex temporal patterns. For instance, the GRU model achieved a Root Mean Squared Error (RMSE) of 369.54 for onions and 210.35 for tomatoes, significantly outperforming the ARIMA model, which recorded RMSE values of 1564.62 and 1298.60, respectively. Furthermore, the Mean Absolute Percentage Error (MAPE) for GRU was notably lower, at 14.59% for onions and 10.58% for tomatoes. These results underscore the efficacy of deep learning approaches in addressing the inherent volatility and nonlinear dynamics of agricultural commodity prices. These findings offer valuable insights for policymakers, traders, and farmers, enabling better market interventions, crop planning, and risk management. The study recommends exploring hybrid models and incorporating external factors like weather data to further enhance forecasting reliability.

## Introduction

Accurate forecasting of agricultural commodity prices is a cornerstone for effective market planning, policy formulation, and risk management in agriculture-driven economies. The agricultural sector, which forms the backbone of economies like India, faces unique challenges due to its exposure to various uncertainties. Price volatility in agricultural commodities, driven by factors such as weather fluctuations, seasonal supply patterns, international trade policies, and global market dynamics, presents a highly complex forecasting challenge. This unpredictability affects a wide range of stakeholders, including farmers striving to make informed decisions about crop selection and marketing, traders managing supply chain risks, and policymakers working to stabilize markets and ensure food security. The complexity of agricultural price forecasting arises from the inherently nonlinear, nonstationary, and stochastic nature of price movements. Traditional methods, such as the Autoregressive Integrated Moving Average (ARIMA) model, have long been the go-to approach for time-series forecasting due to their simplicity and mathematical rigor. These models, while effective for capturing linear relationships in historical data, often fall short in addressing the intricate temporal dependencies and nonlinear patterns inherent in agricultural price dynamics^1,2^. Consequently, reliance solely on traditional statistical models can result in suboptimal forecasts, limiting their utility for real-world decision-making.

In recent years, advancements in machine learning (ML) and deep learning (DL) have paved the way for more robust forecasting methods capable of addressing the limitations of traditional approaches. Machine learning algorithms such as Support Vector Regression (SVR) and Extreme Gradient Boosting (XGBoost) offer enhanced flexibility, enabling them to model multidimensional relationships and nonlinear trends in time-series data. These models have demonstrated considerable success in applications ranging from commodity price forecasting to demand prediction. Meanwhile, deep learning techniques have emerged as a game-changer in the field, driven by their ability to model complex, high-dimensional data and uncover hidden patterns that traditional models often fail to capture. Deep learning models such as Multilayer Perceptron (MLP), Recurrent Neural Networks (RNN), Long Short-Term Memory (LSTM) networks, Gated Recurrent Units (GRU), and Echo State Networks (ESN) have further revolutionized forecasting tasks. These models are particularly well-suited to agricultural price forecasting, as they excel at capturing long-term dependencies, seasonality, and temporal dynamics in data. Their ability to learn from large datasets with minimal human intervention makes them an attractive option for stakeholders seeking to improve forecasting accuracy and reliability.

This study evaluates the effectiveness of these advanced methodologies in forecasting the prices of 23 agricultural commodities using daily wholesale price data from the Agricultural Marketing Information Network (AGMARKNET) portal. Covering a period from January 2010 to June 2024 and spanning 165 markets, this dataset provides a rich basis for analyzing and comparing different predictive models. The commodities are chosen so as to represent various classes of agricultural commodities such as - Spices (Turmeric, Coriander, Dry Chiles, Cumin), Oilseeds (Groundnut, Soyabean, Mustard, Sesamum), Cereals (Wheat, Ragi, Bajra, Maize, Jowar, Paddy), Pulses (Arhar, Bengal Gram, Lentil, Greengram, Kabuli Channa), Vegetables (Onion, Tomato, Potato) and Fiber (Cotton). The performance of stochastic models, machine learning techniques, and deep learning approaches is assessed using standard error metrics such as Root Mean Squared Error (RMSE), Relative Normalized Mean Squared Error (RNMSE), Mean Absolute Error (MAE), and Mean Absolute Percentage Error (MAPE). By examining these models’ predictive capabilities, this research aims to identify the most suitable approach for agricultural price forecasting while also exploring opportunities for model enhancement.

This study contributes to the advancement of agricultural price forecasting research by conducting a rigorous evaluation of traditional statistical methods, machine learning algorithms, and deep learning models across a diverse range of commodities in India, using daily price data spanning over a decade. The novelty of this work lies in its empirical demonstration of the consistent superiority of deep learning models, particularly Long Short-Term Memory (LSTM) networks and Gated Recurrent Units (GRU), across multiple error metrics. Additionally, the study explores the suitability of these models for capturing the complex temporal and nonlinear characteristics of highly volatile agricultural price data. Unlike existing studies, this research utilizes an extensive dataset comprising 23 commodities from 165 markets, enabling a detailed and robust validation of model performance. Furthermore, the study emphasizes the practical implications of improved forecasting accuracy, offering actionable insights for stakeholders such as policymakers, farmers, and traders. It also underscores the potential of hybrid modeling approaches and the incorporation of external variables, such as weather data, to further enhance forecasting reliability and robustness.

The rest of the paper is structured as follows. Section "Literature Review" reviews existing literature on agricultural price forecasting, focusing on the evolution of stochastic models, the rise of machine learning, and the recent impact of deep learning techniques. Section "Methodology" details the methodology, including a description of the dataset, preprocessing steps, and the mathematical underpinnings of each model evaluated in the study. The models include ARIMA, SVR, XGBoost, MLP, LSTM, GRU, and ESN, each explained in terms of their theoretical framework and application to the problem at hand. Section "Results and Discussions" presents the results and discussion, offering a comparative analysis of the models’ performance and providing insights into their strengths and limitations. Finally, Sect. "Conclusion" concludes the paper by summarizing the findings, discussing their practical implications for stakeholders, and suggesting directions for future research, such as the development of hybrid models and the integration of external factors like weather data for further improving forecast accuracy.

## Literature review

The agricultural sector is highly susceptible to price volatility caused by various factors such as weather anomalies, policy changes, and market dynamics^3–5^. Accurate forecasting of agricultural commodity prices is critical for ensuring market stability, supporting farmers’ livelihoods, and enhancing food security. Traditional statistical methods, such as the Autoregressive Integrated Moving Average (ARIMA) model^6^, have long been the foundation of time-series forecasting. Despite their simplicity and interpretability, these models fall short in capturing the nonlinearity and abrupt changes inherent in agricultural prices^7,8^. Machine learning (ML) models such as Support Vector Regression (SVR) and Extreme Gradient Boosting (XGBoost) have demonstrated superior performance in time-series forecasting due to their ability to handle nonlinear relationships and high-dimensional data^9,10^. SVR, based on structural risk minimization, has been particularly effective in mitigating overfitting and handling noisy data. XGBoost, with its ensemble learning approach, has shown remarkable success in commodity price forecasting tasks^3,11^.

While these ML models provide improvements over traditional approaches, their inability to capture long-term temporal dependencies limits their forecasting accuracy in sequential data. This gap has been addressed through the advent of deep learning techniques. Deep learning (DL) models such as Recurrent Neural Networks (RNNs), Long Short-Term Memory (LSTM) networks, and Gated Recurrent Units (GRUs) have transformed time-series forecasting by capturing complex temporal patterns and long-term dependencies^12–15^. Patel^16^ demonstrated the superior performance of LSTM and GRU models in forecasting the volatile prices of vegetables in India, achieving lower error metrics compared to ARIMA and SVR. Echo State Networks (ESNs), introduced by Jaeger^17^, offer an alternative deep learning approach with reduced computational costs. By training only, the output layer while keeping the recurrent layer fixed, ESNs efficiently capture dynamic temporal patterns.

Recent advancements in machine learning have underscored the efficacy of neural networks and Gaussian process regression for modelling nonlinear and complex patterns across various domains. Neural networks have shown remarkable versatility and robustness in capturing intricate dependencies and temporal dynamics, as demonstrated in studies focusing on economic and energy modelling^18,19^. Additionally, their adaptability to diverse datasets and scalability have positioned them as powerful tools for forecasting in financial and agricultural contexts^20–22^. On the other hand, Gaussian process regression provides a probabilistic framework that excels in uncertainty quantification and capturing nonlinear relationships, making it particularly suitable for forecasting tasks in data-scarce or noisy environments^23,24^. Studies in operations management and environmental forecasting have demonstrated its ability to improve prediction reliability and interpretability^25,26^. These findings emphasize the growing relevance of machine learning models in tackling complex real-world problems, thereby motivating the present study’s exploration of their application to agricultural price forecasting.

Hybrid models have emerged as powerful tools for improving forecasting accuracy. The ARIMA-LSTM hybrid model, proposed by Zhang^27^, combines ARIMA’s linear pattern modeling capabilities with LSTM’s strength in capturing nonlinear dependencies. This hybrid approach has shown significant improvements in agricultural price forecasting, as highlighted by Khashei and Bijari^28^. Ensemble methods, such as the Particle Swarm Optimization-based Weighted Ensemble Variance (P-WEV) model, optimize model weights dynamically to leverage the strengths of different forecasting techniques.

Bonawitz^29^ highlighted the potential of Federated Learning (FL) in preserving data privacy while enabling collaborative learning. In the context of agricultural forecasting, FL enables regional models to be trained locally and aggregated at a central server. This approach captures region-specific patterns while leveraging global insights. A recent study by Yang et al.^30^ applied FL to predict crop prices across different markets, demonstrating improved forecasting accuracy and robustness against data heterogeneity. Zhu et al. explored a federated LSTM model for commodity price forecasting, where local LSTM models were trained on market-specific data and their parameters aggregated. This federated approach effectively captured local variations while benefiting from the global model’s generalized learning capabilities. FL also addresses concerns related to data privacy and compliance with regulations such as the General Data Protection Regulation (GDPR), making it an attractive solution for agricultural markets where data confidentiality is paramount^31,32^.

Comparative studies consistently demonstrate that deep learning models outperform traditional and ML models in terms of error metrics such as Root Mean Squared Error (RMSE) and Mean Absolute Percentage Error (MAPE)^4^^16^^33^. Hybrid and ensemble approaches further enhance forecasting performance by integrating the strengths of individual models. Federated learning, though relatively new in this domain, shows great promise in improving forecasting accuracy while addressing data privacy and scalability concerns. Studies by Yang et al.^30^ and Zhu et al. underline the potential of FL to revolutionize agricultural price forecasting by enabling collaborative, decentralized model training.

To summarize previous studies have extensively explored traditional statistical models like ARIMA and machine learning approaches such as SVR and XGBoost to forecast agricultural commodity prices. However, these methods often fall short in handling the nonlinear and volatile nature of price data. Deep learning techniques, including LSTM and GRU, have demonstrated superior performance in capturing complex temporal patterns and long-term dependencies, but prior research typically focused on limited datasets or fewer commodities. Gaps remain in leveraging external variables like weather data and hybridizing models for improved accuracy and robustness. This study addresses these gaps by analyzing 23 commodities across 165 markets over 14 years, empirically validating the superior performance of LSTM and GRU while advocating for hybrid models and external factor integration, thus contributing to more reliable and actionable forecasting solutions​.

## Methodology

### Data description

The daily wholesale data from 2010-02-26 to 2024-06-11 for 23 different commodities across 165 markets have been collected from the AGMARKNET Portal (http://agmarknet.gov.in/*).* These 23 commodities include 3 vegetables (Potato, Onion and Tomato), 4 oilseeds (Mustard, Sesamum, Groundnut and Soybean), 5 pulses (Greengram, Kabuli Channa, Lentil, Bengal Gram and Arhar), 4 spices (Turmeric, Dry Chilies, Cumin and Coriander) and 6 cereals (Paddy, Jowar, Maize, Wheat, Ragi and Bajra) and Cotton which is a fiber crop. Different classes of commodity have been selected for their different levels of volatility. The selection of the markets and commodities in each class was based on their maximum share and representation.

The commodity with the highest variation, as measured by the coefficient of variation (CV), is Onion, with a CV of approximately 0.765. None of the commodities exhibit normality based on the Jarque-Bera test, as all have probabilities indicating non-normality. The descriptive statistics can be seen in Table 1.

**Table 1:** Descriptive statistics of wholesale prices of agricultural commodities.

Commodity	Mean	Median	Maximum	Minimum	Std Dev	CV	Skewness	Kurtoises
**Turmeric**	11,421.15	11,000.00	110,000.00	4,250.00	3,849.98	0.34	6.16	148.67
**Mustard**	3,852.34	3,500.00	13,500.00	1,875.00	1,012.08	0.26	1.57	5.85
**Paddy**	1,337.87	1,360.00	2,395.00	692.00	279.96	0.21	0.27	0.11
**Greengram**	6,935.56	7,800.00	9,400.00	2,000.00	1,929.28	0.28	(0.97)	(0.53)
**Cotton**	4,908.85	4,650.00	10,750.00	1,705.00	1,501.09	0.31	0.81	1.16
**Dry Chilies**	6,151.24	5,750.00	15,000.00	1,300.00	2,287.94	0.37	0.91	0.55
**Kabuli Chana**	6,063.75	5,350.00	14,000.00	0.00	2,140.33	0.35	0.74	(0.10)
**Sesamum**	9,081.71	9,000.00	15,500.00	4,000.00	2,744.18	0.30	0.33	(0.89)
**Cumin**	14,428.96	13,000.00	61,500.00	5,240.00	5,607.34	0.39	3.87	20.83
**Soybean**	4,160.33	4,300.00	9,200.00	1,900.00	1,120.67	0.27	0.67	0.46
**Potato**	856.51	810.00	3,800.00	240.00	496.75	0.58	1.58	3.34
**Groundnut**	4,535.68	4,255.00	8,955.00	0.00	1,282.69	0.28	0.51	(0.62)
**Jowar**	2,159.88	2,000.00	5,200.00	300.00	693.58	0.32	0.83	0.51
**Lentil**	3,699.05	3,420.00	7,015.00	1,000.00	1,116.75	0.30	0.71	(0.38)
**Maize**	1,748.04	1,853.00	2,100.00	850.00	311.02	0.18	(0.93)	0.06
**Arhar**	4,916.88	4,600.00	11,000.00	2,105.00	1,642.93	0.33	1.15	0.89
**Onion**	1,634.92	1,300.00	10,000.00	320.00	1,250.26	0.77	2.37	8.55
**Tomato**	1,509.14	1,300.00	8,100.00	200.00	986.25	0.65	2.79	13.98
**Bengal Gram**	4,119.08	4,122.50	8,915.00	1,750.00	1,088.28	0.26	0.55	2.44
**Wheat**	1,734.52	1,830.00	4,000.00	935.00	417.43	0.24	(0.02)	(0.29)
**Ragi**	1,587.81	1,600.00	3,350.00	680.00	569.48	0.36	0.21	(0.70)
**Coriander**	5,717.86	5,540.00	12,005.00	1,975.00	1,989.62	0.35	0.67	0.21
**Bajra**	1,569.38	1,460.00	3,500.00	675.00	442.15	0.28	0.82	0.08

Note: Author’s own representation.

To ensure the validity of our time series modeling approach, we examined the stationarity of the price series using two complementary tests: the Augmented Dickey-Fuller (ADF) and Kwiatkowski-Phillips-Schmidt-Shin (KPSS) tests. The ADF test evaluates the null hypothesis that a unit root is present in the time series (indicating non-stationarity). The test is based on the following regression model which is seen in Eq. 1:

1 $$\varDelta\:{y}_{t}=\alpha+\beta\:t+\gamma\:{y}_{t-1}+{\delta}_{1}\varDelta\:{y}_{t-1}+{\delta}_{21}\varDelta\:{y}_{t-2}+...+{\delta}_{p}\varDelta\:{y}_{t-p}+{\epsilon}_{t}$$

where $\:{y}_{t}$ represents the time series, $\:\varDelta\:{y}_{t}$ = $\:{y}_{t}$ − $\:{y}_{t-1}$, t is a time trend, and $\:p$ is the lag order. The null hypothesis of a unit root corresponds to $\:\gamma\:$ = 0, while the alternative hypothesis of stationarity requires $\:\gamma\:$ < 0. Complementing the ADF test, we employed the KPSS test which reverses the hypotheses. The KPSS test assumes stationarity under the null hypothesis and tests for the presence of a unit root in the alternative. The test statistic is based on the residuals from the regression as seen in Eq. 2.1:

2 $${y}_{t}=\alpha+\beta\:t+{r}_{t}+{\epsilon}_{t}$$

where $\:{r}_{t}$ follows a random walk: $\:{r}_{t}$ = $\:{r}_{t-1}$ + $\:{u}_{t}$, with $\:{u}_{t}$ ~ i.i.d.(0, $\:{\sigma\:}_{u}^{2}$). The KPSS test statistic is defined as seen in Eq. 2.2:

2.2 $$KPSS=\frac{{\sum}_{t=1}^{T}{S}_{t}^{2}}{{T}^{2}{\widehat{\sigma}}_{\epsilon}^{2}}$$

where $\:{S}_{t}=$ ${\sum}_{j=1}^{T}{\widehat{\epsilon}}_{j}$, ${\widehat{\epsilon}}_{t}$ are the residuals, and $\:{\widehat{\sigma}}_{\epsilon}^{2}$ is a consistent estimate of the long-run variance of ${\epsilon}_{t}$.

The results for both the stationarity tests can be seen in Table 2.

**Table 2:** ADF and KPSS stationarity test results.

Commodity	ADF Statistics	ADF *P* Value	ADF Stationary	KPSS Statistic	KPSS *P* Value	KPSS Stationary	Interpretation
**Turmeric**	−3.072102459	0.028687807	TRUE	0.810118684	0.01	FALSE	Conflicting results: ADF indicates stationary, KPSS indicates non-stationary.
**Mustard**	−2.210559617	0.202435273	FALSE	3.206036378	0.01	FALSE	Series is non-stationary (both tests agree)
**Paddy**	−0.302921615	0.925089524	FALSE	14.46263202	0.01	FALSE	Series is non-stationary (both tests agree)
**Greengram**	−1.040387679	0.738250402	FALSE	3.091695665	0.01	FALSE	Series is non-stationary (both tests agree)
**Cotton**	−2.155569088	0.222764471	FALSE	4.906140972	0.01	FALSE	Series is non-stationary (both tests agree)
**Dry Chillies**	−2.57420075	0.098447538	FALSE	5.63095073	0.01	FALSE	Series is non-stationary (both tests agree)
**Kabuli Channa**	−2.218664231	0.199541287	FALSE	2.319520883	0.01	FALSE	Series is non-stationary (both tests agree)
**Seasamum**	−2.197552436	0.20713498	FALSE	1.860341729	0.01	FALSE	Series is non-stationary (both tests agree)
**Cumin**	−3.636380713	0.005102408	TRUE	2.630377179	0.01	FALSE	Conflicting results: ADF indicates stationary, KPSS indicates non-stationary.
**Soyabean**	−2.25857184	0.185677545	FALSE	3.294392627	0.01	FALSE	Series is non-stationary (both tests agree)
**Potato**	−3.043796982	0.031001842	TRUE	2.897407828	0.01	FALSE	Conflicting results: ADF indicates stationary, KPSS indicates non-stationary.
**Groundnut**	−2.154928733	0.223008262	FALSE	8.503065282	0.01	FALSE	Series is non-stationary (both tests agree)
**Jowat**	−2.55230524	0.103260809	FALSE	3.283243999	0.01	FALSE	Series is non-stationary (both tests agree)
**Lentil**	−1.162947857	0.689328173	FALSE	4.018158718	0.01	FALSE	Series is non-stationary (both tests agree)
**Maize**	−3.269542777	0.016296032	TRUE	3.446192871	0.01	FALSE	Conflicting results: ADF indicates stationary, KPSS indicates non-stationary.
**Arhar**	−0.485939313	0.894731262	FALSE	2.775224225	0.01	FALSE	Series is non-stationary (both tests agree)
**Onion**	−5.456214455	2.58E-06	TRUE	0.315071991	0.1	TRUE	Series is stationary (both tests agree)
**Tomato**	−5.104295503	1.37E-05	TRUE	0.42840487	0.064911694	TRUE	Series is stationary (both tests agree)
**Bengalgram**	−2.67250253	0.078918591	FALSE	1.311960998	0.01	FALSE	Series is non-stationary (both tests agree)
**Wheat**	−0.920343225	0.781199305	FALSE	6.517613509	0.01	FALSE	Series is non-stationary (both tests agree)
**Ragi**	−1.394234212	0.585037167	FALSE	5.950177776	0.01	FALSE	Series is non-stationary (both tests agree)
**Coriander**	−2.780925513	0.061050503	FALSE	2.473213306	0.01	FALSE	Series is non-stationary (both tests agree)
**Bajra**	−2.656292151	0.08191178	FALSE	4.777727023	0.01	FALSE	Series is non-stationary (both tests agree)

Note: Author’s own representation.

The unit root test results indicate that most commodity price series are non-stationary, as confirmed by both the ADF and KPSS tests. Only **onion and tomato** are clearly stationary, suggesting their prices revert to a mean over time. Several commodities, including **mustard**,** paddy**,** greengram**,** cotton**,** and wheat**, are non-stationary according to both tests, meaning they exhibit persistent trends and require transformations like differencing for further analysis. A few commodities, such as **turmeric**,** cumin**,** potato**,** and maize**, show conflicting results—where the ADF test suggests stationarity, but KPSS indicates non-stationarity—implying potential trend stationarity or structural breaks. Overall, most series require additional preprocessing to ensure they meet stationarity assumptions for time series modeling.

### Machine learning models

#### Autoregressive integrated moving average (ARIMA) model

The Autoregressive Integrated Moving Average (ARIMA) model is a significant tool in predictive modeling, providing enhanced flexibility in fitting time-series data by combining both autoregressive and moving average components. This combination results in the ARMA (p, q) model (Eq. 3), a concept introduced by Box and Pierce in 1970. The fundamental assumption of this model is that the time series should be time-invariant, meaning it remains constant or stationary over time.

The ARMA (p, q) model can be expressed as:

3 $${y}_{t}={\varphi}_{1}{y}_{\left(t-1\right)}+{\varphi}_{2}{y}_{\left(t-2\right)}+...+{\varphi}_{p}{y}_{\left(t-p\right)}+{\theta}_{1}{\varepsilon}_{\left(t-1\right)}+{\theta}_{2}{\varepsilon}_{\left(t-2\right)}+...+{\theta}_{q}{\varepsilon}_{\left(t-q\right)}$$

Using the backshift operator B, this can be equivalently written as:

$$\varphi\left(B\right){y}_{t}=\theta\left(B\right){\varepsilon}_{t}$$

where,

$$\varphi\left(B\right)=1-{\varphi}_{1}B-{\varphi}_{2}{B}^{2}-...-{\varphi}_{p}{B}^{p}$$

$$\theta\left(B\right)=1-{\theta}_{1}B-{\theta}_{2}{B}^{2}-...-{\theta}_{q}{B}^{q}$$

To handle nonstationary time-series data, ARMA models can be extended by incorporating a method called “differencing,” as described by Makridakis et al. in 1982. This extension results in the ARIMA (p, d, q) model (Eq. 4), which is formulated as:

4 $$\varphi\left(B\right){\left(1-B\right)}^{d}{y}_{t}=\theta\left(B\right){\varepsilon}_{t}$$

where $\:{\epsilon\:}_{t}$ are identically and independently distributed (IID) as N(0, $\:{\sigma\:}^{2}$), and the integration parameter ‘d’ is a non-negative integer.

The algorithm for ARIMA forecasting can be seen below:

##### Algorithm

ARIMA for Commodity Price Forecasting.

Input: Historical price data P, order parameters (p, d, q), forecast horizon H.

Output: Forecasted prices F.

1. Perform Augmented Dickey-Fuller test to check stationarity.
2. If data is non-stationary, apply differencing (d times) until stationary.
3. Select optimal (p, d, q) using AIC/BIC.
4. Fit ARIMA model with selected (p, d, q).
5. Forecast next H time steps.
6. Return forecasted prices F.

#### Support vector regression (SVR)

Support Vector Regression (SVR) (Eq. 5) is a machine learning technique for non-linear estimation, following the principle of structural risk minimization (Valiant, 1984). It aims to minimize the upper limit of generalization error. The SVR model can be expressed as:

5 $$f(x,z)=f(x,\alpha,\alpha^\wedge)=\sum(\alpha_{i}-\alpha_{i}^\wedge)\phi(x,xi)+b$$

where $\:f\left(x,z\right)$ is the model’s output, and the kernel function $\phi\:\left(.\right)$ transforms the non-linear dataset into a higher-dimensional feature space, assuming linearity.

A key feature of SVR is the ε-insensitive loss function, which introduces a margin of tolerance around the predicted output. Errors within this margin, defined by a parameter ϵ\epsilonϵ, are ignored, while deviations beyond it are penalized. This allows SVR to create a robust regression model that focuses on significant patterns without being overly sensitive to noise or outliers in the data. By tuning ϵ\epsilonϵ, users can control the trade-off between precision and generalization, making SVR particularly effective for datasets with moderate noise levels.

SVR models leverage kernels to handle nonlinear relationships in the data. A kernel function maps input features into a higher-dimensional space where a linear relationship can be established. Popular kernel functions include the linear, polynomial, and radial basis function (RBF) kernels, with the RBF kernel being widely used due to its ability to capture complex patterns. The choice of kernel and its associated hyperparameters significantly influences SVR’s performance and flexibility.

The algorithm for running SVR is shown below:

##### Algorithm

SVR for Commodity Price Forecasting.

Input: Historical price data P, kernel function K, hyperparameters C and epsilon.

Output: Forecasted prices F.

1. Preprocess data: Normalize prices P.
2. Define feature set X and target variable Y.
3. Split dataset into training and test sets.
4. Train SVR model with kernel K, regularization parameter C, and epsilon.
5. Predict prices on test set.
6. Evaluate performance using RMSE, MAE.
7. Return forecasted prices F.

#### Multilayer perceptron (MLP)

**Fig. 1:**
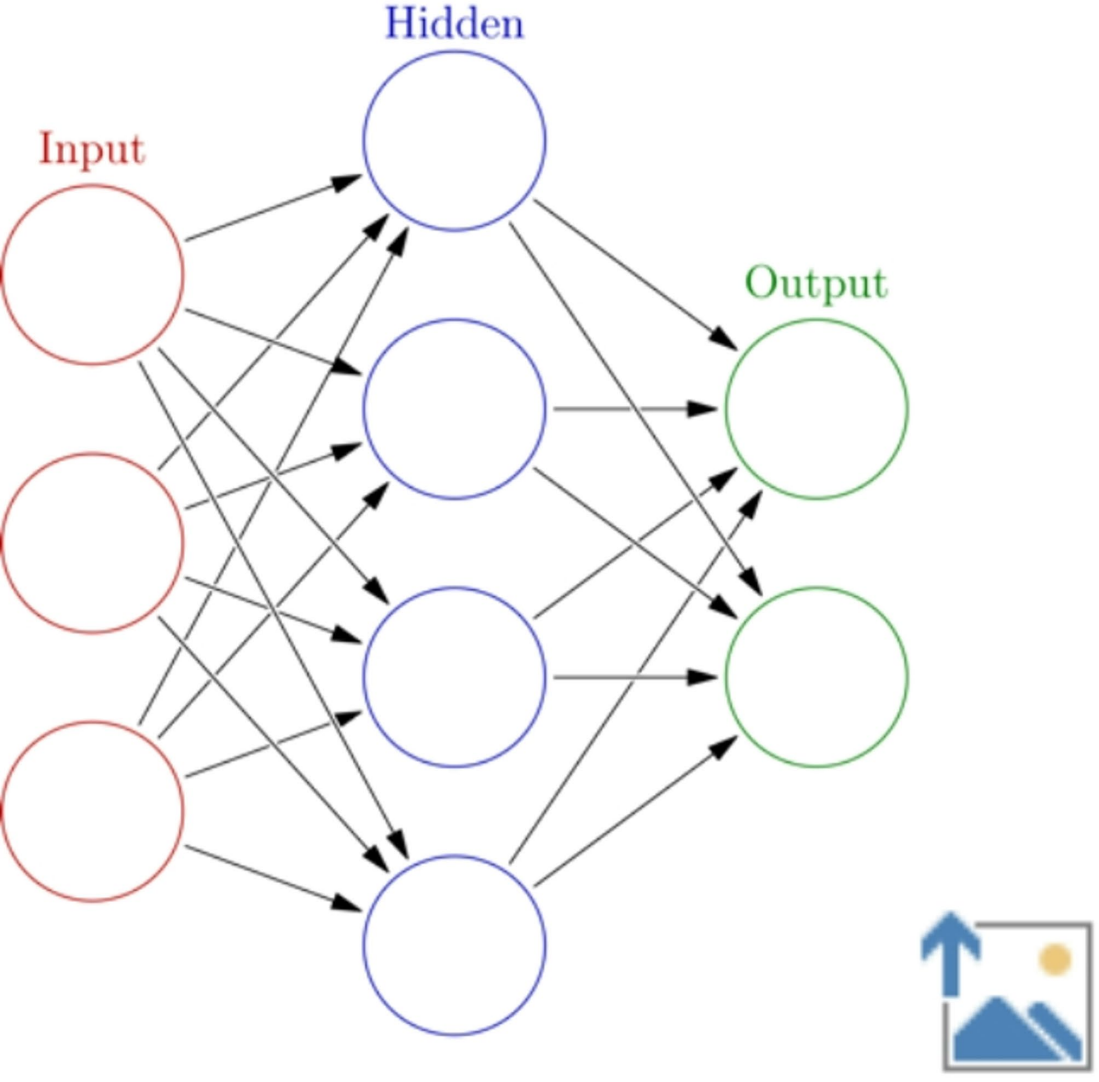
Architecture of Multilayer Perceptron and Artificial Neuron. *Colored Neural Network* by Sam John, CC BY-SA 4.0.

The Multilayer Perceptron (MLP) (Eq. 6) is a foundational model in the field of artificial neural networks. It was first introduced by Marvin Minsky and Seymour Papert in their 1969 book “Perceptrons,” although the term “MLP” and its widespread application in practical tasks came later. An MLP consists of multiple layers of neurons, including an input layer, one or more hidden layers, and an output layer. Each neuron in a layer is connected to every neuron in the subsequent layer. MLPs use backpropagation, an algorithm popularized by Rumelhart, Hinton, and Williams in their seminal 1986 paper, for training. This algorithm adjusts weights to minimize the error between the predicted and actual outputs. The architecture of a MLP model can be seen in Fig. 1.

The mathematical representation of a neuron in an MLP is given by:

6 $$Y=\sigma\left({\sum}_{i=1}^{N}{w}_{i}{x}_{i}+b\right)$$

where $\:Y$ is the output, $\:{x}_{i}$ are the input values, $\:{w}_{i}$ are the weights, $\:b$ is the bias term, and $\:\sigma\:$ is the activation function, typically a non-linear function like ReLU or sigmoid.

MLP is run using this algorithm:

##### Algorithm

MLP for Commodity Price Forecasting.

Input: Historical price data P, number of hidden layers L, neurons per layer N, activation function A.

Output: Forecasted prices F.

1. Normalize price data P.
2. Define input features X and target variable Y.
3. Split dataset into training and test sets.
4. Initialize MLP with L hidden layers, N neurons per layer, activation function A.
5. Train model using backpropagation and Adam optimizer.
6. Predict prices on test set.
7. Evaluate performance using RMSE, MAE.
8. Return forecasted prices F.

#### Recurrent neural networks (RNN)

**Fig. 2:**
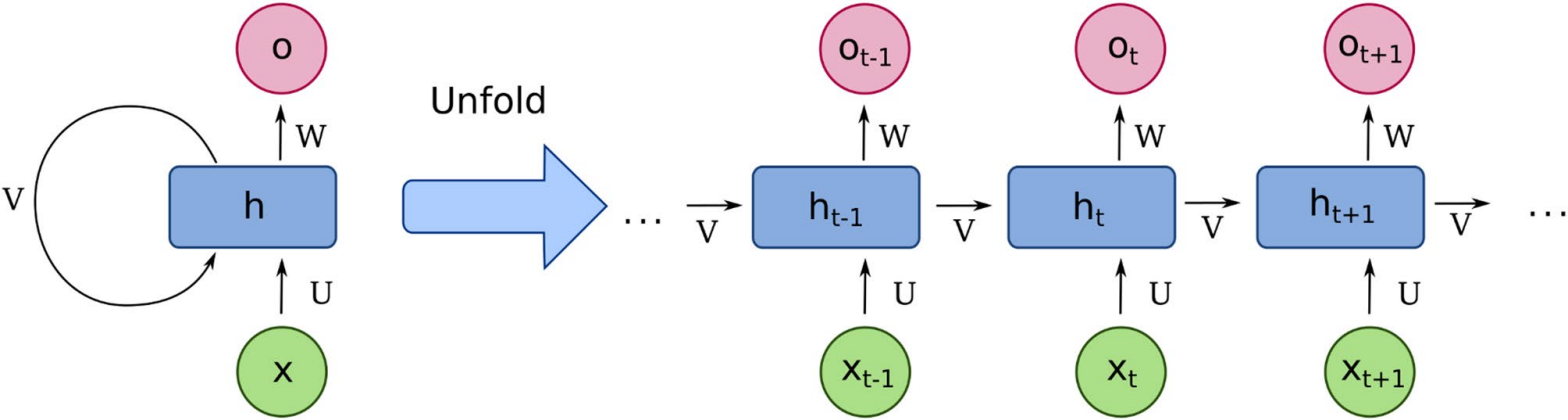
Architecture of Recurrent Neural Network. *Fourier Transform Visualization* by Tomruen, CC0 1.0.

Recurrent Neural Networks (RNNs) (Eq. 7) are a specialized type of neural network in which the output from a previous step serves as the input for the current step. In contrast to conventional neural networks, where inputs and outputs are treated independently, RNNs are designed to handle sequences, making them ideal for tasks like predicting the next word in a sentence, which relies on information from previous words. This distinctive feature introduces the concept of a “hidden layer” or “memory state” that retains information about past inputs. The concept of RNNs was first introduced by Elman in 1990. The architecture of a RNN model can be seen in Fig. 2.

The defining characteristic of RNNs is their ability to process input sequences of arbitrary length. At each time step, the network takes the current input and the hidden state from the previous step to compute the new hidden state. This hidden state acts as a dynamic memory, preserving context from earlier inputs. Mathematically, the hidden state is computed as:

7 $$\:{h}_{t+1}={f}_{H}\left({W}_{U}\cdot\:{x}_{t}+{{W}_{V}\cdot\:{h}_{t}}_{\:}\right){o}_{t+1}={f}_{O}\left({W}_{W}\cdot\:{h}_{t+1}\right)$$

These equations incorporate three key connection weight matrices: $\:{W}_{U}$, $\:{W}_{V}$, and $\:{W}_{W}$​. The network’s hidden and output units are characterized by activation functions $\:{f}_{H}$ and $\:{f}_{O}$​.

The algorithm for RNN is as below:

##### Algorithm

RNN for Commodity Price Forecasting.

Input: Historical price data P, number of time steps T, number of hidden units H.

Output: Forecasted prices F.

1. Normalize and reshape data into sequences of length T.
2. Define RNN architecture with H hidden units.
3. Train RNN using stochastic gradient descent.
4. Predict prices for next time steps.
5. Evaluate model performance.
6. Return forecasted prices F.

#### Long Short-Term memory (LSTM) networks

**Fig. 3:**
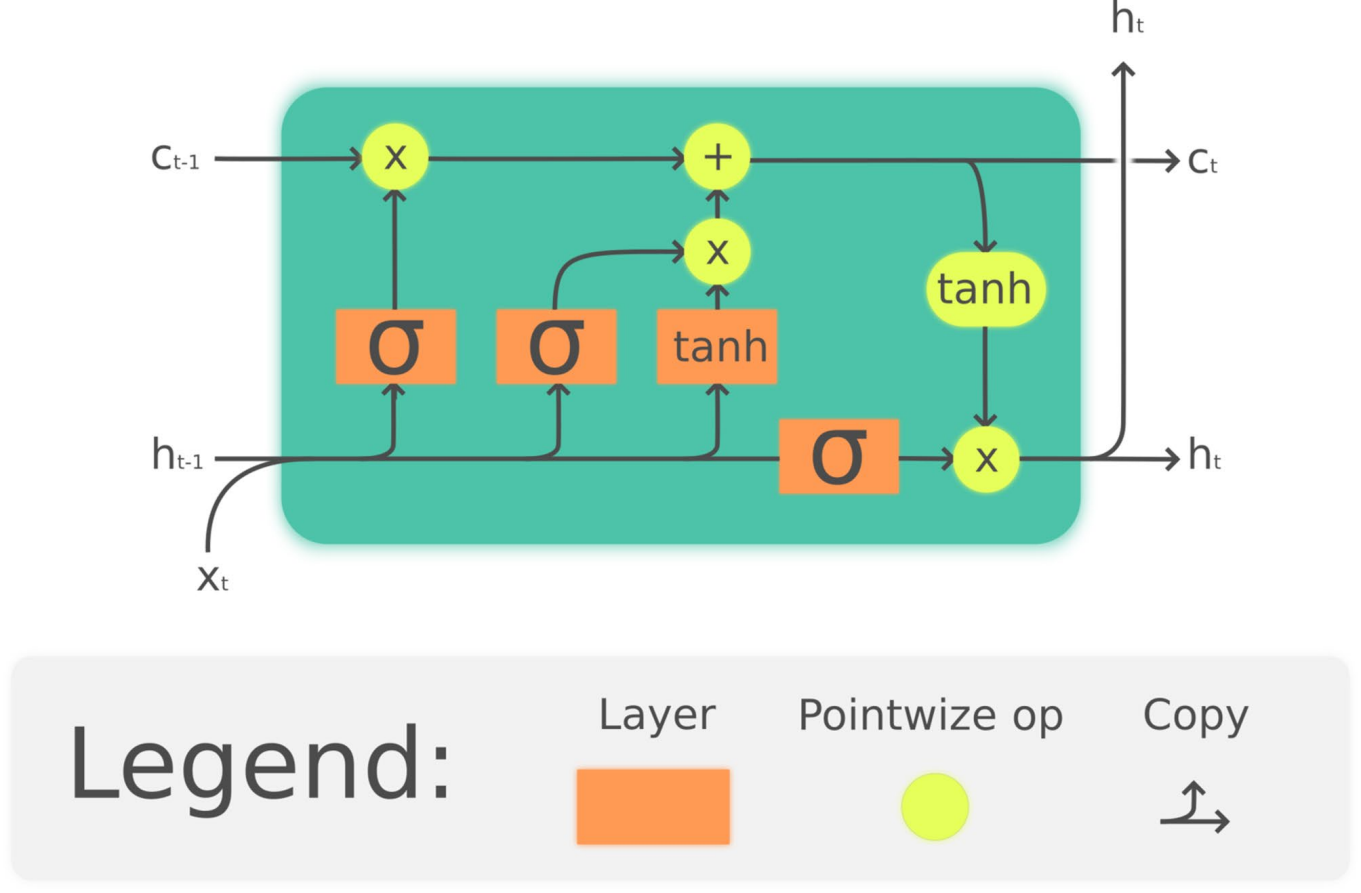
Architecture of a single LSTM cell. *LSTM Cell* by fdelrosso, CC BY-SA 4.0.

The Long Short-Term Memory (LSTM) network is an advanced type of Recurrent Neural Network (RNN) that effectively addresses the vanishing gradient problem, which limits traditional RNNs in learning long-term dependencies. LSTM achieves this through its unique architecture comprising three key gates: the forget gate, input gate, and output gate. These gates regulate the flow of information, enabling the network to retain relevant information over extended sequences while discarding irrelevant patterns. The forget gate discards unnecessary information from the previous time step, ensuring that the model focuses only on the important aspects of the data. The input gate determines what new information from the current time step should be added to the memory, while the output gate decides what information to pass on to the next time step. This gating mechanism empowers LSTM to handle complex, nonlinear, and sequential data effectively, making it particularly suitable for forecasting agricultural commodity prices, which exhibit strong temporal dependencies and volatility. The architecture can be seen in Fig. 3. The equations that define a LSTM cell are the Eqs. 8.1 to 8.6.

The mathematical formulation of LSTM cells involves:

8.1 $$\:{f}_{t}=\sigma\:\left({W}_{f}\cdot\:\left[{h}_{t-1},{x}_{t}\right]+{b}_{f}\right)$$

8.2 $$\:{i}_{t}=\sigma\:\left({W}_{i}\cdot\:\left[{h}_{t-1},{x}_{t}\right]+{b}_{i}\right)$$

8.3 $$\:{o}_{t}=\sigma\:\left({W}_{o}\cdot\:\left[{h}_{t-1},{x}_{t}\right]+{b}_{o}\right)$$

8.4 $${C}_{t}^\wedge=tanh\left({W}_{C}\cdot\:\left[{h}_{t-1},{x}_{t}\right]+{b}_{C}\right)$$

8.5 $${C}_{t}={f}_{t}\cdot{C}_{t-1}+{i}_{t}\cdot{C}_{t}^\wedge$$

8.6 $$\:ht={o}_{t}\cdot\:tanh\left({C}_{t}\right)$$

where $\:{f}_{t}$, $\:{i}_{t}$, and $\:{o}_{t}$ are the forget, input, and output gates, respectively; $\:{C}_{t}$ is the cell state; $\:{h}_{t}$ is the hidden state; $\:{x}_{t}$ is the input; and $\:W$ and $\:b$ are the weights and biases.

The daily wholesale price data for 23 agricultural commodities was carefully preprocessed to ensure compatibility with the LSTM model. The first step was normalization, where the price data was scaled to a range between 0 and 1. This preprocessing step is critical to ensure stable and efficient convergence during model training and to prevent larger numerical values from dominating the learning process. Next, a sliding window approach was employed to structure the data into overlapping time-series windows. Each window consisted of 1000 consecutive time steps, allowing the model to learn both short-term fluctuations and long-term trends effectively. Finally, the dataset was divided into an 80% training set and a 20% testing set, ensuring the model was validated on unseen data to assess its generalization ability. This systematic data preparation process ensured that the LSTM model had access to clean, well-structured input for learning temporal patterns.

The LSTM model was implemented using Python and the TensorFlow/Keras library. The architecture was carefully designed to balance performance and computational efficiency. The input layer was configured to accept sequences of 1000 time steps, with each step representing a single price point. This was followed by a single LSTM layer with 128 units, chosen after extensive hyperparameter tuning to ensure the model’s capacity to learn complex patterns without overfitting. To further reduce the risk of overfitting, a dropout layer with a rate of 0.2 was included. This layer randomly deactivated 20% of neurons during training, encouraging the network to learn robust representations. The final dense output layer, consisting of a single neuron with a linear activation function, was used to predict the price for the next time step. This simple yet effective architecture ensured that the LSTM model could handle the nonlinearities and temporal dependencies inherent in the price data.

The training process for the LSTM model was designed to optimize its performance while ensuring stability and efficiency. The Mean Squared Error (MSE) loss function was used, as it penalizes large deviations between predicted and actual values, making it suitable for regression tasks like price forecasting. The Adam optimizer was employed to minimize the loss, offering adaptive learning rates that accelerate convergence and improve training efficiency. The model was trained in batches of size 64 over 50 epochs, with 20% of the training data set aside for validation. This validation step allowed the model’s performance to be monitored during training and helped prevent overfitting by ensuring it generalized well to unseen data. The training process was further supported by early stopping mechanisms to halt training when validation loss stopped improving. These carefully designed training procedures ensured that the LSTM model achieved high accuracy and robustness, particularly for volatile commodities like onions and tomatoes.

To run LSTM, the following algorithm is used:

##### Algorithm

LSTM for Commodity Price Forecasting.

Input: Historical price data P, sequence length T, LSTM units U.

Output: Forecasted prices F.

1. Normalize price data P.
2. Convert data into overlapping sequences of length T.
3. Define LSTM model with U units.
4. Train model using backpropagation through time (BPTT).
5. Predict future prices.
6. Evaluate model performance.
7. Return forecasted prices F.

#### Gated recurrent unit (GRU) networks

**Fig. 4:**
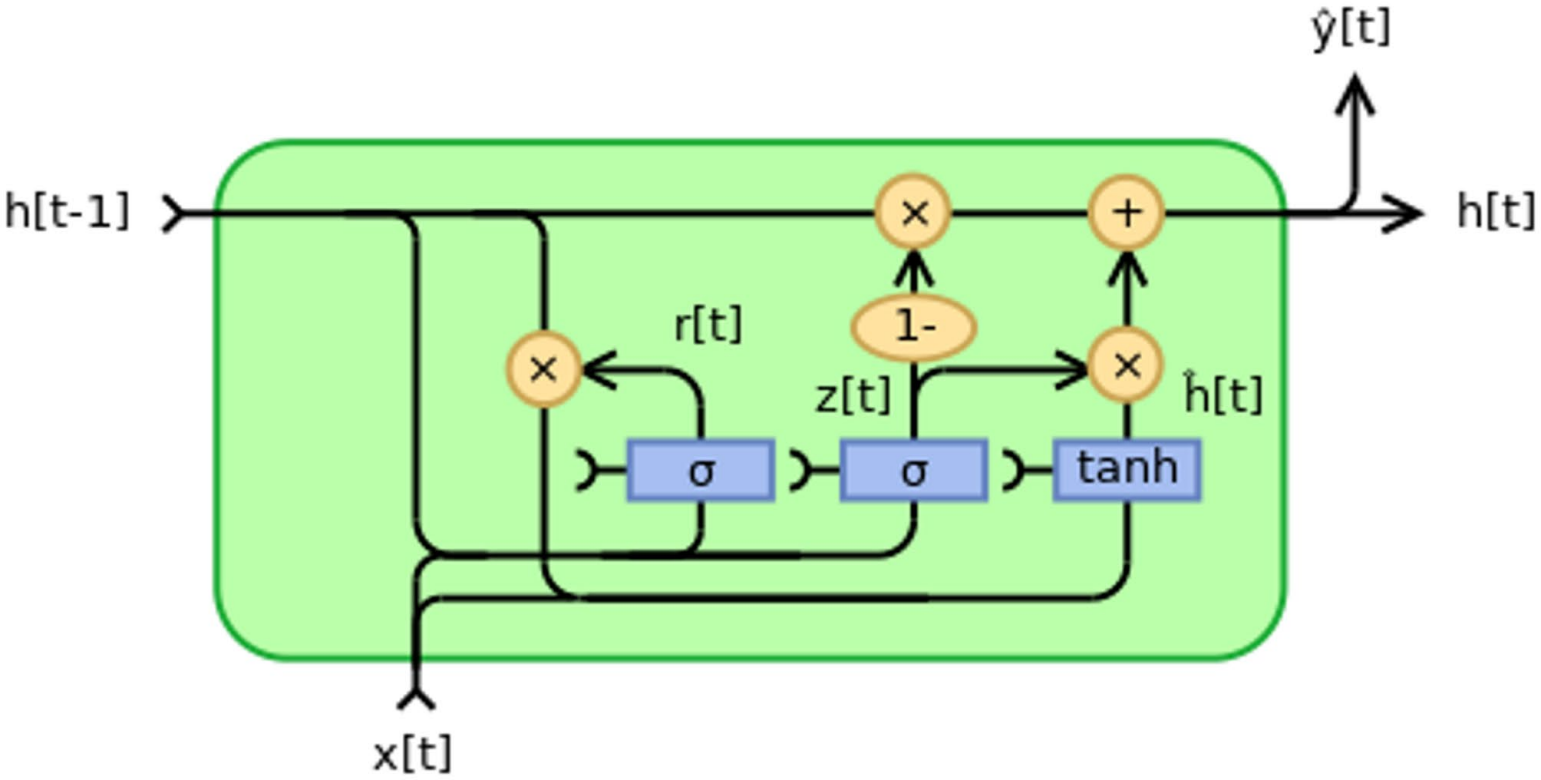
Architecture of a single GRU cell. *Gated Recurrent Unit (Type 1)* by fdelrosso, CC BY-SA 4.0.

The Gated Recurrent Unit (GRU) (Fig. 4) is a simplified version of the Long Short-Term Memory (LSTM) (3.2.5) network, designed to address the limitations of traditional Recurrent Neural Networks (RNNs). Like LSTM, GRU is well-suited for capturing long-term dependencies in sequential data, making it a powerful tool for forecasting agricultural commodity prices. The GRU architecture replaces the multiple gates in LSTM with two primary gates: the reset gate and the update gate. These gates regulate the flow of information within the network, enabling it to selectively remember or forget information over time. The reset gate determines the degree to which the previous hidden state contributes to the current hidden state, allowing the network to disregard irrelevant information. The update gate, on the other hand, decides how much of the previous information needs to be carried forward to the next time step. This streamlined gating mechanism reduces the computational complexity of GRU compared to LSTM while maintaining similar performance in many tasks. GRU’s lightweight architecture is particularly beneficial when computational efficiency is a priority.

The mathematical representation of GRU cells includes:

9.1 $$\:zt=\sigma\:\left({W}_{z}\cdot\:\left[{h}_{t-1},{x}_{t}\right]+{b}_{z}\right)$$

9.2 $$\:{r}_{t}=\sigma\:\left({W}_{r}\cdot\:\left[{h}_{t-1},{x}_{t}\right]+{b}_{r}\right)$$

9.3 $${h}_{t}^\wedge=tanh\left(W\cdot\left[{r}_{t}\cdot{h}_{t-1},{x}_{t}\right]+b\right)$$

9.4 $${h}_{t}=\left(1-{z}_{t}\right)\cdot{h}_{t-1}+{z}_{t}\cdot{h}_{t}^\wedge$$

where $\:{z}_{t}$ and $\:{r}_{t}$ are the update and reset gates, respectively; $\:{h}_{t}$ is the hidden state; $\:{x}_{t}$ is the input; and $\:W$ and $\:b$ are the weights and biases.

The daily wholesale price data for 23 agricultural commodities was meticulously prepared to ensure it was suitable for the GRU model. The first step in the process was normalization, where the price values were scaled to a range between 0 and 1. This transformation improved the stability of the training process by preventing large numerical values from dominating the learning process and ensuring all features contributed equally. Following normalization, the data was restructured using a sliding window approach, which involved splitting the dataset into overlapping sequences of 1000 time steps. Each sequence represented a continuous block of historical prices, enabling the GRU model to learn both short-term variations and long-term dependencies within the data. This approach allowed the model to extract temporal patterns effectively and handle the inherent volatility in agricultural price data. Lastly, the dataset was divided into two subsets: 80% for training and 20% for testing. This train-test split ensured that the model’s performance was evaluated on unseen data, providing a robust measure of its generalization ability. These preprocessing steps collectively ensured that the data was clean, well-structured, and ready for training the GRU model.

The GRU model was implemented using Python and TensorFlow/Keras. The architecture was carefully designed to balance simplicity and predictive accuracy. The input layer was configured to accept sequences of 1000 time steps, where each step represented a single price point. The core of the model was a single GRU layer with 128 units, selected based on hyperparameter tuning to optimize performance without introducing unnecessary complexity. Unlike LSTM, GRU does not require a separate cell state, which simplifies its architecture and reduces memory requirements. To prevent overfitting, a dropout layer with a rate of 0.2 was added, randomly deactivating neurons during training to encourage the model to learn robust patterns. The dense output layer, with one neuron and a linear activation function, was used to predict the next price in the sequence. This straightforward architecture leveraged GRU’s efficiency while maintaining high accuracy.

The GRU model was trained using the Mean Squared Error (MSE) loss function, which penalizes large deviations between predicted and actual values. The Adam optimizer was employed for optimization, offering adaptive learning rates that improve convergence speed and stability. The model was trained with a batch size of 64 over 50 epochs, and 20% of the training data was used for validation. The validation step allowed for real-time monitoring of the model’s performance during training and ensured that the model did not overfit to the training data. Early stopping mechanisms were also implemented, halting the training process if the validation loss did not improve for a specified number of epochs. This approach ensured that the model was not over-trained and could generalize well to new data. By the end of training, the GRU model demonstrated strong predictive accuracy, particularly for highly volatile commodities such as onions and tomatoes, where it achieved consistently low RMSE and MAPE values.

To run GRU, the following algorithm is used:

##### Algorithm

GRU for Commodity Price Forecasting.

Input: Historical price data P, sequence length T, GRU units U.

Output: Forecasted prices F.

1. Normalize and reshape data into sequences of length T.
2. Define GRU architecture with U units.
3. Train GRU using backpropagation through time.
4. Predict prices for the next time steps.
5. Evaluate model performance.
6. Return forecasted prices F.

#### Extreme gradient boosting (XGBoost)

Extreme Gradient Boosting (XGBoost) is an ensemble learning algorithm that builds multiple decision trees in a sequential manner to improve the overall predictive accuracy. XGBoost enhances the standard gradient boosting algorithm by incorporating regularization techniques, parallel processing, and efficient handling of missing values, making it particularly effective for structured data and tabular datasets. Unlike deep learning models like LSTM or GRU, XGBoost focuses on modeling nonlinear patterns through decision tree ensembles, which partition the feature space to optimize the prediction performance. Each tree in XGBoost learns from the residual errors of the previous trees, progressively refining the model’s accuracy. This iterative process is particularly useful for datasets with moderate nonlinearity and less temporal complexity, such as agricultural price data for moderately volatile commodities. XGBoost’s efficiency and scalability make it a strong candidate for forecasting tasks, especially where computational resources are limited.

XGBoost is based on the principle of gradient boosting, where the model sequentially minimizes a loss function by adding decision trees as weak learners. The objective function in XGBoost comprises two parts: the training loss and a regularization term. The objective function is defined as:

10.1 $$\text{L}(\phi)=\sum(i=1 \:\text{to}\:\text{n})1(y_i,\hat{y}_i)+\sum(k=1\:\text{to}\:K)\Omega(f_k)$$

where l(y_i_, ŷ_i_) is the loss function measuring the difference between the actual value y_i_ and the predicted value ŷ_i_, and Ω(f_k_) is the regularization term for the k-th tree, defined as Ω(f_k_) = γT + ½λ‖w‖². Here, T is the number of leaves in the tree, λ is the L2 regularization term for leaf weights, and γ controls the minimum loss reduction required to perform a split. This formulation balances model accuracy and complexity, ensuring robustness and preventing overfitting.

At each iteration t, XGBoost refines the predictions by adding a new tree f_t_(x) to the model, such that the prediction is updated as:

10.2 $$\hat{y}_i^{t}=\hat{y}_i^{t-1}+f_t(x_i)$$

where $\hat{y}_i^{(t-1)}$ is the prediction from the previous t − 1 trees and f_t_(x_i_) is the output of the current tree. To efficiently minimize the loss, XGBoost uses a second-order Taylor expansion to approximate the objective function:

10.3 $$\text{L}^{t}\approx\sum(i=1\:\text{to}\:\text{n})[g_if_t(x_i)+1/2h_{i}f_t^2(x_i)]+\Omega(f_t)$$

Here, $g_i=\partial1(y_i,\hat{y_i})/\partial\hat{y}_i$ is the first-order gradient (error) and $h_i=\partial^21(y_i,\hat{y_i})/\partial\hat{y}_i^2$ is the second-order gradient (curvature). These gradients enable efficient optimization of the objective function by guiding the updates in the tree-building process.

The optimal weights for the leaves of a tree are computed as:

10.4 $$w_j=-\sum(i\in\text{I}_j)g_i/\sum(i\in\text{I}_j)h_i+\lambda$$

where $\text{I}_j$ is the set of data points in leaf j. The gain from splitting a node is calculated as:

10.5 $$\begin{aligned}\text{Gain}&=1/2[(\sum(i\in I_1)g_i)^2/(\sum(i\in I_1)h_i\\& \quad+\lambda)+(\sum(i\in I_r)g_i)^2/(\sum(i\in I_r)h_i+\lambda)\\& \quad-(\sum(i\in I_p)g_i)^2/(\sum(i\in I_p)h_i+\gamma)\end{aligned}$$

Here, I_1_, I_r_, and I_p_ are the sets of data points in the left child, right child, and parent nodes, respectively, and γ is the regularization term for split complexity. This formulation ensures that splits are only performed when they result in meaningful improvements to the model.

The final prediction for a data point x_i_ is computed as the sum of the outputs from all K trees:

10.6 $$\hat{y}_i=\sum(k=1\:\text{to}\:\text{K})f_k(x_i)$$

The XGBoost model was configured to maximize its ability to capture the nonlinearities and patterns in the price data. The input features consisted of historical price sequences, with each feature corresponding to a specific time step. Key hyperparameters were tuned to enhance the model’s performance:

- Number of Trees: The number of decision trees was set to 500 to ensure the model had sufficient capacity to capture complex relationships without overfitting.
- Learning Rate: A learning rate of 0.1 was chosen to balance the speed of training and the model’s ability to converge to an optimal solution.
- Maximum Tree Depth: The depth of each tree was set to 6, ensuring the model captured sufficient complexity while avoiding overfitting.
- Regularization: L1 and L2 regularization terms were included to penalize overly complex models and improve generalization.
- Early Stopping: Early stopping was implemented based on the validation loss to halt training when performance plateaued, reducing unnecessary computational expense.

This configuration allowed XGBoost to efficiently learn patterns in the agricultural price data while maintaining scalability and robustness.

The XGBoost model was trained using the Mean Squared Error (MSE) as the objective function, which minimized the squared difference between predicted and actual values. During training, the model incrementally added decision trees, each learning to predict the residual errors of the previous trees. This iterative process allowed XGBoost to refine its predictions continuously, improving accuracy with each step. The model was trained with a batch size of 128 over 100 boosting rounds, with early stopping triggered if validation loss did not improve after 10 consecutive rounds. Hyperparameter tuning was conducted using grid search to identify the optimal combination of tree depth, learning rate, and regularization parameters. Once trained, the model was evaluated on the test set using metrics such as RMSE, MAE, and MAPE.

The algorithm for running XGBoost is as below:

##### Algorithm

XGBoost for Commodity Price Forecasting.

Input: Historical price data P, feature set X, target Y, hyperparameters.

Output: Forecasted prices F.

1. Preprocess and engineer features from P.
2. Split dataset into training and test sets.
3. Train XGBoost model with hyperparameters.
4. Predict prices on test set.
5. Evaluate model using RMSE, MAE.
6. Return forecasted prices F.

#### Echo state networks (ESN)

**Fig. 5:**
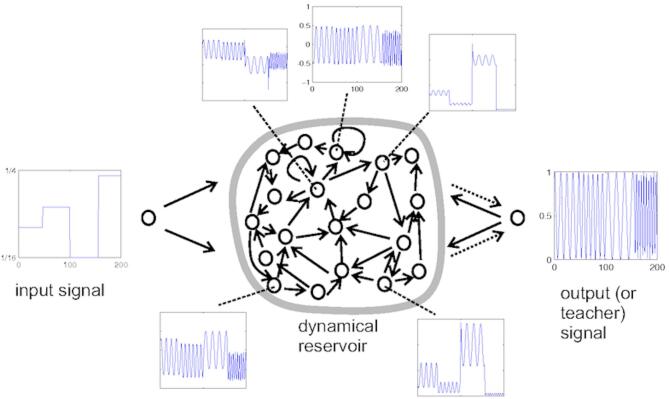
Architecture of Echo State Networks. *Frequency Generator Schema* by Arnold Reinhold, CC BY-SA 3.0.

Echo State Networks (ESNs) (Fig. 5) are a specialized type of Recurrent Neural Network (RNN) designed to efficiently handle time-series prediction tasks by leveraging their unique architecture. Introduced by Jaeger in 2001, ESNs address the computational challenges typically associated with training RNNs by keeping the internal recurrent structure, referred to as the “reservoir,” fixed. Instead of training the entire network, ESNs focus on optimizing only the output layer, significantly reducing training time while maintaining the ability to capture complex temporal dynamics.

The reservoir, a large and sparsely connected set of neurons, serves as the core of the ESN. It is initialized randomly and remains unchanged during training, ensuring that it provides a diverse range of dynamic responses to input sequences. These responses are mapped to the output layer, where the actual learning occurs. This separation between the untrained reservoir and the trainable output weights enables ESNs to efficiently process sequential data without the risk of overfitting or requiring extensive computational resources.

One of the defining characteristics of ESNs is their reliance on the “echo state property.” This property ensures that the influence of past inputs fades over time, allowing the network to maintain stability and focus on recent information. Mathematically, the reservoir state at time is computed as a function of the current input, the previous reservoir state, and a set of fixed weights. The output is then derived as a linear combination of these reservoir states using trainable weights.

The ESN can be mathematically described as:

11.1 $$\:{x}_{t}=tanh\left({W}_{\:}\cdot\:{u}_{t}+W{\cdot\:x}_{t-1}\right)$$

11.2 $$\:{y}_{t}={W}_{out}\cdot\:{x}_{t}$$

where $\:{x}_{t}$ is the state of the reservoir at time $\:t$, $\:{u}_{t}$ is the input, $\:{W}_{}$ is the input weight matrix, $\:W$ is the reservoir weight matrix, and $\:{W}_{out}$ is the output weight matrix that is trained. The reservoir states $\:{x}_{t}$ capture the temporal dynamics, and the readout $\:{y}_{t}$ provides the final prediction.

ESN is run using the algorithm below:

##### Algorithm

ESN for Commodity Price Forecasting.

Input: Historical price data P, reservoir size R, spectral radius S, sparsity parameter Sp.

Output: Forecasted prices F.

1. Normalize data P.
2. Create an ESN with reservoir size R, spectral radius S, and sparsity Sp.
3. Train readout layer using ridge regression.
4. Predict future prices.
5. Evaluate performance.
6. Return forecasted prices F.

### Prediction accuracy

The prediction accuracy of different models compared based on four error measures. Namely, Root Mean Squared Error (RMSE) (Eq. 12), Relative Normalized Mean Squared Error (RNMSE) (Eq. 13), Mean Absolute Error (MAE) (Eq. 14) and Mean Absolute Percentage Error (MAPE) (Eq. 15). The formulas for each are given below.

12 $$\:RMSE=\sqrt{\frac{1}{n}{\sum}_{i=1}^{n}{\left({y}_{i}-\hat{{y}_{i}}\right)}^{2}}$$

13 $$\:RNMSE=\frac{\sqrt{\frac{1}{n}{\sum}_{i=1}^{n}{\left({y}_{i}-\widehat{{y}_{i}}\right)}^{2}}}{\frac{1}{n}{\sum}_{i=1}^{n}{y}_{i}}$$

14 $$\:MAE=\frac{1}{n}{\sum}_{i=1}^{n}\left|{y}_{i}-\widehat{{y}_{i}}\right|$$

15 $$\:MAPE=\frac{100}{n}{\sum}_{i=1}^{n}\left|\frac{{y}_{i}-\widehat{{y}_{i}}}{{y}_{i}}\right|$$

**RMSE** is sensitive to large errors due to the squaring. We chose RMSE because we want to heavily penalize models for large prediction misses (which, in commodity markets, could correspond to failing to predict a price surge or crash). A lower RMSE means the model’s predictions on average deviate less in a quadratic sense from actual prices. However, RMSE is scale-dependent (in units of the commodity’s price) and can be dominated by a few outliers. To counter scale-dependence, **RNMSE** is reported too.

**MAE** is the average absolute error. It is more interpretable in terms of actual price units (e.g., rupees per quintal) and is less skewed by outliers than RMSE. We examine MAE to understand typical error magnitude without the extra penalty on outliers. In some cases, we found RMSE and MAE giving different rankings for models if one model occasionally had big errors. Reporting both helps cross-check robustness: if a model has a much higher RMSE than MAE relative to another, it implies occasional large misses.

**MAPE** is the mean absolute percent error. We include MAPE to gauge error relative to the actual price level in percentage terms. This is crucial when comparing forecast accuracy across commodities with very different price scales. MAPE normalizes errors, allowing apples-to-apples comparison.

By using all four metrics, we satisfy multiple criteria: RMSE and RNMSE for emphasizing big errors, MAE for general reliability, and MAPE for relative error. We also considered alternatives like Theil’s U statistic and Mean Absolute Scaled Error (MASE). Theil’s U can benchmark against a no-change forecast, and MASE would allow comparison to naive seasonal forecasts, but we ultimately chose RMSE, MAE, MAPE as they are widely understood and cover the needs of our evaluation. Notably, these metrics align with those commonly used in the literature, ensuring our results can be compared to other studies. Where one model is best in RMSE but another in MAPE, it provides insight: perhaps one model handles scale better (lower MAPE) but has occasional large errors (hence higher RMSE). This nuanced evaluation helps determine the truly “best” model depending on what error profile is more important for stakeholders (e.g., avoiding large shocks vs. overall consistency).

## Results and discussions

### Accuracy results

The analysis evaluated the performance of various machine learning models on predicting prices of different agricultural commodities, including MLP, GRU, LSTM, RNN, XGBoost, Linear Regression, SVR, ESN, and ARIMA. The models ran on a window size of 1000 with a 80% training data and 20% test data split. The results indicated that recurrent neural network models, particularly GRU and RNN, consistently delivered superior accuracy across most commodities, as evidenced by their lower MAE and MAPE values. These models effectively captured the temporal dependencies in agricultural price data, making them highly suitable for forecasting tasks.

Conversely, while XGBoost and traditional models like ARIMA showed reasonable performance, they often lagged behind the neural network models, especially in handling complex, non-linear patterns in the data. Despite its simplicity, Linear Regression performed competitively in some cases, highlighting that simpler models can sometimes yield good results. The findings suggest prioritizing GRU and RNN models for practical forecasting applications due to their robustness and reliability while also advocating for further research into hybrid models and the integration of external factors to enhance prediction accuracy. The performance of these models for a few selected commodities is illustrated in Table 3 for RMSE, Table 4 for RNMSE, Table 5 for MAE, and Table 6 for MAPE.

**Table 3:** Prediction performance based on RMSE.

Commodity	GRU	ARIMA	Linear Regression	SVR	XGBoost	LSTM	ESN	MLP	RNN
**Onion**	374.97	1,564.62	1,575.05	1,524.07	421.83	372.16	969.79	1,373.12	369.54
**Jowar**	213.97	1,775.75	1,773.42	1,699.03	514.34	185.36	603.36	1,726.92	184.71
**Mustard**	295.75	3,254.08	3,296.00	3,183.31	445.50	178.73	884.75	2,173.38	173.70
**Lentil**	310.94	3,047.08	3,096.70	2,967.26	390.08	287.76	1,133.75	2,755.84	287.59
**Cumin**	707.36	8,951.23	6,022.58	6,772.06	947.11	704.02	3,382.93	2,916.79	670.22
**Greengram**	636.05	5,709.28	5,728.47	5,676.19	3,204.65	435.55	3,399.52	3,603.88	388.51
**Bajra**	155.77	1,210.62	1,290.63	1,087.12	212.78	151.70	301.10	2,249.57	149.28
**Soybean**	443.80	3,281.84	3,318.20	3,213.18	1,074.27	414.57	1,813.40	2,470.00	390.38
**Dry Chillies**	324.13	5,258.67	5,371.19	5,088.94	1,635.66	332.30	2,026.39	3,581.79	1,410.10
**Wheat**	36.55	1,319.53	1,368.62	1,213.86	499.91	36.31	671.53	2,355.08	33.96
**Kabuli Chana**	630.99	5,055.60	5,133.74	4,930.01	1,171.53	624.11	1,974.92	8,439.36	620.30
**Ragi**	94.55	1,122.52	1,168.19	1,011.22	374.18	97.55	840.83	1,657.48	92.25
**Sesamum**	846.07	8,084.79	8,108.42	8,054.39	1,793.93	736.78	3,142.42	3,495.54	745.95
**Turmeric**	770.77	7,041.91	7,120.23	6,906.77	4,656.50	962.00	3,848.56	3,748.62	884.54
**Bengalgram**	335.12	4,056.97	4,099.44	3,999.65	1,165.47	282.12	1,469.47	1,578.21	270.68
**Arhar**	447.00	4,239.67	4,261.64	4,169.64	654.61	405.17	1,824.46	2,477.63	399.08
**Tomato**	238.98	1,298.60	1,301.77	1,252.57	338.26	212.37	639.61	1,148.21	210.35
**Paddy**	86.95	641.38	785.83	185.36	249.74	82.90	444.07	462.37	105.12
**Coriander**	658.22	5,160.26	5,226.10	5,022.80	875.81	610.01	2,131.09	4,511.02	607.80
**Maize**	66.21	1,475.45	1,492.09	1,446.40	369.65	51.30	536.28	3,119.58	44.18
**Potato**	85.21	561.68	604.55	486.48	191.65	79.80	438.40	1,239.64	72.03
**Cotton**	799.07	3,999.35	4,080.76	3,896.59	884.65	736.18	1,753.61	5,441.72	705.47
**Groundnut**	268.70	3,544.83	3,688.18	3,299.59	297.20	263.48	1,261.25	2,733.99	268.56

Note: Author’s representation of the results.

**Table 4:** Prediction performance based on RNMSE.

Commodity	GRU	ARIMA	Linear Regression	SVR	XGBoost	LSTM	ESN	MLP	RNN
**Onion**	0.08	0.32	0.32	0.31	0.09	0.08	0.20	0.28	0.08
**Jowar**	0.08	0.67	0.67	0.64	0.20	0.07	0.23	0.65	0.07
**Mustard**	0.10	1.09	1.11	1.07	0.15	0.06	0.30	0.73	0.06
**Lentil**	0.07	0.65	0.67	0.64	0.08	0.06	0.24	0.59	0.07
**Cumin**	0.06	0.98	0.98	0.96	0.08	0.06	0.28	1.71	0.06
**Greengram**	0.09	0.83	0.84	0.83	0.47	0.06	0.50	0.53	0.06
**Bajra**	0.11	0.82	0.88	0.74	0.14	0.10	0.20	1.53	0.12
**Soybean**	0.06	0.45	0.45	0.44	0.15	0.06	0.25	0.34	0.06
**Dry Chilies**	0.04	0.71	0.72	0.68	0.22	0.04	0.27	5.31	0.27
**Wheat**	0.03	1.26	1.31	1.16	0.48	0.03	0.64	2.25	0.03
**Kabuli Chana**	0.07	0.58	0.59	0.57	0.13	0.07	0.23	0.97	0.10
**Ragi**	0.06	0.68	0.70	0.61	0.23	0.06	0.51	1.00	0.06
**Sesamum**	0.09	0.85	0.85	0.84	0.19	0.08	0.33	0.37	0.08
**Turmeric**	0.05	0.70	0.71	0.69	0.30	0.06	0.24	4.48	0.06
**Bengalgram**	0.05	0.57	0.57	0.56	0.16	0.04	0.21	0.22	0.04
**Arhar**	0.06	0.52	0.53	0.51	0.08	0.05	0.23	0.31	0.05
**Tomato**	0.07	0.39	0.39	0.38	0.10	0.06	0.19	0.35	0.06
**Paddy**	0.07	0.48	0.59	0.14	0.19	0.06	0.33	0.35	0.09
**Coriander**	0.07	0.51	0.52	0.50	0.09	0.06	0.21	0.45	0.06
**Maize**	0.06	1.24	1.25	1.21	0.31	0.04	0.45	0.17	0.04
**Potato**	0.05	0.31	0.33	0.26	0.10	0.04	0.24	0.67	0.04
**Cotton**	0.14	0.71	0.72	0.69	0.16	0.13	0.31	0.96	0.14
**Groundnut**	0.04	0.57	0.59	0.53	0.05	0.04	0.20	0.44	0.07

Note: Author’s representation of the results.

**Table 5:** Prediction performance based on MAE.

Commodity	GRU	ARIMA	Linear Regression	SVR	XGBoost	LSTM	ESN	MLP	RNN
**Onion**	217.73	1,234.25	1,247.43	1,182.42	254.78	229.70	831.18	1,105.52	208.91
**Jowar**	153.54	1,712.90	1,710.49	1,633.22	347.98	126.52	476.35	1,392.30	122.07
**Mustard**	250.05	3,213.44	3,255.89	3,141.76	276.10	119.29	774.82	1,706.69	112.57
**Lentil**	204.73	2,959.36	3,010.42	2,877.10	290.46	189.68	1,001.54	2,175.81	190.08
**Cumin**	495.79	11,820.75	11,892.88	11,639.57	698.76	484.45	2,975.42	15,330.74	461.18
**Greengram**	440.89	5,397.27	5,417.57	5,362.26	2,600.66	267.32	2,835.41	3,047.46	209.66
**Bajra**	112.35	1,184.99	1,266.62	1,058.50	156.89	110.91	235.80	1,756.24	110.50
**Soybean**	203.58	3,204.50	3,241.73	3,134.15	976.50	197.83	1,743.67	2,143.67	166.94
**Dry Chilies**	205.62	5,002.99	5,121.14	4,824.28	974.15	225.85	1,733.99	21,921.44	1,349.24
**Wheat**	18.50	1,283.76	1,334.17	1,174.89	368.63	17.76	543.20	1,809.21	13.83
**Kabuli Chana**	335.67	4,857.05	4,937.39	4,727.67	602.50	298.75	1,698.49	6,141.63	287.79
**Ragi**	60.70	1,052.88	1,101.44	933.31	313.72	69.37	729.75	1,317.89	56.70
**Sesamum**	479.38	7,662.55	7,687.48	7,630.48	1,506.88	350.22	2,826.58	2,755.61	321.37
**Turmeric**	284.84	10,408.15	10,491.21	10,264.67	958.19	615.11	3,396.69	4,020.36	404.65
**Bengalgram**	198.72	3,798.54	3,843.87	3,737.26	828.70	166.33	1,153.61	1,315.66	147.28
**Arhar**	261.00	4,008.85	4,032.09	3,934.73	528.62	226.52	1,724.80	2,003.36	213.65
**Tomato**	164.28	1,138.77	1,142.38	1,085.98	218.62	139.30	551.11	924.91	122.83
**Paddy**	63.72	614.12	763.74	153.92	198.10	57.27	377.72	393.04	75.16
**Coriander**	454.73	4,806.70	4,877.32	4,658.83	661.62	400.05	1,827.07	3,650.24	396.12
**Maize**	38.82	1,452.23	1,469.13	1,422.71	285.91	28.93	463.76	235.71	20.79
**Potato**	36.18	445.18	498.18	345.55	152.50	28.73	400.25	949.11	23.71
**Cotton**	607.76	3,868.44	3,952.55	3,762.11	624.51	540.55	1,509.15	3,649.10	522.44
**Groundnut**	183.23	3,461.20	3,607.77	3,209.74	213.50	176.88	1,057.70	2,118.01	183.21

Note: Author’s representation of the results.

**Table 6:** Prediction performance based on MAPE.

Commodity	GRU	ARIMA	Linear Regression	SVR	XGBoost	LSTM	ESN	MLP	RNN
**Onion**	16.17	92.66	94.14	86.82	21.42	18.65	94.64	119.08	14.59
**Jowar**	8.54	93.07	92.93	88.41	25.07	6.89	33.19	82.93	6.46
**Mustard**	7.83	97.52	98.84	95.29	9.27	3.70	26.06	53.99	3.46
**Lentil**	7.24	96.38	98.13	93.56	10.63	6.64	37.70	76.67	6.57
**Cumin**	4.11	98.60	99.22	97.06	6.34	4.05	27.51	130.24	3.84
**Greengram**	9.77	99.28	99.71	98.55	68.90	5.95	73.87	78.14	4.40
**Bajra**	9.15	87.78	94.08	78.01	13.23	8.92	20.87	137.94	8.83
**Soybean**	5.56	97.88	99.05	95.66	31.99	5.53	56.68	68.11	4.51
**Dry Chilies**	3.97	95.62	98.12	91.84	22.36	5.38	43.19	505.96	28.01
**Wheat**	1.36	89.63	93.33	81.62	32.37	1.36	46.16	134.89	1.02
**Kabuli Chana**	1.50	38.5	17.3	72.8	1.62	1.53	1.76	3.20	1.53
**Ragi**	4.86	88.46	93.02	77.22	34.61	5.96	80.01	137.11	4.43
**Sesamum**	6.32	99.41	99.76	98.95	24.65	4.67	44.14	43.59	4.26
**Turmeric**	2.89	98.43	99.32	96.91	11.21	6.51	39.33	43.28	4.02
**Bengalgram**	4.86	97.75	99.08	95.94	28.81	4.35	38.12	43.08	3.69
**Arhar**	6.04	98.12	98.73	96.17	12.91	5.05	45.70	53.20	4.78
**Tomato**	14.81	92.17	92.57	86.33	30.29	13.65	74.62	107.80	10.58
**Paddy**	5.77	53.19	66.81	13.97	20.08	5.16	37.76	38.33	6.48
**Coriander**	8.85	97.38	99.03	93.93	17.39	7.96	49.14	87.27	7.85
**Maize**	3.01	97.31	98.49	95.26	23.26	2.16	36.09	16.85	1.50
**Potato**	5.93	67.79	78.77	47.18	37.42	4.95	95.34	194.56	4.00
**Cotton**	17.49	96.24	98.49	93.39	19.96	15.30	46.90	103.54	14.77
**Groundnut**	58.2	49.2	25.0	90.8	5.82	5.75	7.66	3.69	5.72

Note: Author’s representation of the results.

### Results statistical significance

The Diebold-Mariano (DM) test is a statistical method used to compare the predictive accuracy of two competing forecast models. It determines whether the difference in forecast errors is statistically significant. In this section, the DM test is conducted to compare the predictive performance of the deep learning model against another forecasting model, assessing whether the differences in their forecast errors are statistically significant. The test is based on the loss differential, which represents the difference in forecasting errors of the two models. The DM test statistic is calculated as shown in Eq. 16. The DM test results for RMSE, RNMSE, MAE and MAPE between a few of the machine learning models can be seen in Tables 7, 8, 9 and 10 respectively. While the p-values for the respective tests can be seen in Tables 11, 12, 13 and 14 respectively. Extremely small p-values been capped at *1E-300*.

16 $$\:DM=\frac{\stackrel{-}{d}}{\sqrt{\frac{2\pi\:\widehat{{f}_{d}}\left(0\right)}{T}}}$$

where:

$\:\stackrel{-}{d}=\frac{1}{T}{\sum\:}_{t=1}^{T}{d}_{t}$ - is the mean loss difference,

$\:\:{d}_{t}=\:g\left({e}_{\left\{1,t\right\}}\right)-\:g\left({e}_{\left\{2,t\right\}}\right)$- represents the loss differential at time *t*,

$\:{\left\{f\right\}}_{d\left(0\right))}$ - is an estimator of the long-run variance of $\:{d}_{t}$,

$\:T$ - is the number of observations.

**Table 7:** DM values on RMSE.

Model 1	Model 2	Onion	Paddy	Soybean	Tomato	Wheat
GRU	LSTM	−26.724067	−150.97258	−113.54771	−17.584945	−468.61274
GRU	SVR	0.37166818	−109.08007	45.7420929	5.02972961	56.4187096
GRU	XGBoost	39.0855203	−14.840515	169.702067	16.5638971	272.86799
LSTM	SVR	1.29520461	−4.7614724	47.923897	5.50951223	77.1061655
LSTM	XGBoost	44.3006813	125.472613	173.035052	17.8200625	294.619689
MLP	GRU	−52.016998	−6.4134749	−161.36097	−50.943095	−219.20316
MLP	LSTM	−57.544871	−150.22609	−164.31472	−55.320828	−240.92812
MLP	SVR	−13.898817	−114.41626	−27.850771	−8.248109	−145.47036
MLP	XGBoost	−4.3475299	−23.632464	−2.7035124	−6.0460899	−11.8719
XGBoost	SVR	−14.240847	−99.716911	−27.911904	−9.4542975	−107.78134

Note: Author’s representation of the results.

**Table 8:** DM values on RNMSE.

Model 1	Model 2	Onion	Paddy	Soybean	Tomato	Wheat
GRU	LSTM	−26.724067	−150.97258	−113.54771	−17.584945	−468.61274
GRU	SVR	0.37166818	−109.08007	45.7420929	5.02972961	56.4187096
GRU	XGBoost	39.0855203	−14.840515	169.702067	16.5638971	272.86799
LSTM	SVR	1.29520461	−4.7614724	47.923897	5.50951223	77.1061655
LSTM	XGBoost	44.3006813	125.472613	173.035052	17.8200625	294.619689
MLP	GRU	−52.016998	−6.4134749	−161.36097	−50.943095	−219.20316
MLP	LSTM	−57.544871	−150.22609	−164.31472	−55.320828	−240.92812
MLP	SVR	−13.898817	−114.41626	−27.850771	−8.248109	−145.47036
MLP	XGBoost	−4.3475299	−23.632464	−2.7035124	−6.0460899	−11.8719
XGBoost	SVR	−14.240847	−99.716911	−27.911904	−9.4542975	−107.78134

Note: Author’s representation of the results.

**Table 9:** DM values on MAE.

Model 1	Model 2	Onion	Paddy	Soybean	Tomato	Wheat
GRU	LSTM	−296956.89	−144.35393	−1,000,000	−10267.666	−1000
GRU	SVR	8.34183592	−112.81628	32.8126734	19.1291975	55.3321621
GRU	XGBoost	15.9000945	−14.580169	100.706009	23.1449495	595.034586
LSTM	SVR	9.06004867	−0.4388706	33.8543081	19.5850503	75.3228482
LSTM	XGBoost	16.5652507	120.822581	102.764109	23.5866684	660.056217
MLP	GRU	−16.751692	−6.9479121	−96.789886	−27.278711	−512.64425
MLP	LSTM	−17.434233	−135.65898	−98.748937	−27.772318	−563.06443
MLP	SVR	−14.838783	−126.46095	−29.635727	−11.980707	−212.90577
MLP	XGBoost	−1.8080477	−24.31047	−2.3626131	−5.2728112	−32.206148
XGBoost	SVR	−14.072562	−104.73541	−29.866444	−11.378778	−106.70783

Note: Author’s representation of the results.

**Table 10:** DM values on MAPE.

Model 1	Model 2	Onion	Paddy	Soybean	Tomato	Wheat
GRU	LSTM	−26.724067	−150.97258	−113.54771	−17.584945	−630.54385
GRU	SVR	0.37166818	−109.08007	45.7420929	5.02972961	67.3596766
GRU	XGBoost	39.0855203	−14.840515	169.702067	16.5638971	365.768711
LSTM	SVR	1.29520461	−4.7614724	47.923897	5.50951223	95.7885252
LSTM	XGBoost	44.3006813	125.472613	173.035052	17.8200625	384.158103
MLP	GRU	−52.016998	−6.4134749	−161.36097	−50.943095	−1143.183
MLP	LSTM	−57.544871	−150.22609	−164.31472	−55.320828	−1249.1727
MLP	SVR	−13.898817	−114.41626	−27.850771	−8.248109	−182.91895
MLP	XGBoost	−4.3475299	−23.632464	−2.7035124	−6.0460899	−35.636685
XGBoost	SVR	−14.240847	−99.716911	−27.911904	−9.4542975	−100.69532

Note: Author’s representation of the results.

**Table 11:** P values for the DM test on RMSE.

Model 1	Model 2	Onion	Paddy	Soybean	Tomato	Wheat
GRU	LSTM	2.58E-71	1.00E-300	3.40E-224	8.39E-45	1.00E-300
GRU	SVR	0.71049441	1.00E-300	1.22E-126	9.73E-07	1.05E-218
GRU	XGBoost	1.72E-101	8.40E-48	4.05E-269	2.10E-41	1.00E-300
LSTM	SVR	0.19659493	2.03E-06	2.31E-131	9.39E-08	2.27E-279
LSTM	XGBoost	3.37E-112	1.00E-300	2.64E-271	1.40E-45	1.00E-300
MLP	GRU	2.54E-126	1.70E-10	1.85E-263	2.54E-129	1.00E-300
MLP	LSTM	2.18E-135	1.00E-300	1.70E-265	4.04E-137	1.00E-300
MLP	SVR	5.17E-32	1.00E-300	3.86E-80	1.13E-14	1.00E-300
MLP	XGBoost	2.10E-05	1.49E-111	0.00731173	5.76E-09	8.36E-29
XGBoost	SVR	4.01E-33	1.00E-300	2.51E-80	3.40E-18	1.00E-300

Note: Author’s representation of the results.

**Table 12:** P values for the DM test on RNMSE.

Model 1	Model 2	Onion	Paddy	Soybean	Tomato	Wheat
GRU	LSTM	2.58E-71	1.00E-300	3.40E-224	8.39E-45	1.00E-300
GRU	SVR	0.71049441	1.00E-300	1.22E-126	9.73E-07	1.05E-218
GRU	XGBoost	1.72E-101	8.40E-48	4.05E-269	2.10E-41	1.00E-300
LSTM	SVR	0.19659493	2.03E-06	2.31E-131	9.39E-08	2.27E-279
LSTM	XGBoost	3.37E-112	1.00E-300	2.64E-271	1.40E-45	1.00E-300
MLP	GRU	2.54E-126	1.70E-10	1.85E-263	2.54E-129	1.00E-300
MLP	LSTM	2.18E-135	1.00E-300	1.70E-265	4.04E-137	1.00E-300
MLP	SVR	5.17E-32	1.00E-300	3.86E-80	1.13E-14	1.00E-300
MLP	XGBoost	2.10E-05	1.49E-111	0.00731173	5.76E-09	8.36E-29
XGBoost	SVR	4.01E-33	1.00E-300	2.51E-80	3.40E-18	1.00E-300

Note: Author’s representation of the results.

**Table 13:** P values for the DM test on MAE.

Model 1	Model 2	Onion	Paddy	Soybean	Tomato	Wheat
GRU	LSTM	1.00E-300	1.00E-300	1.00E-300	1.00E-300	1.00E-300
GRU	SVR	7.75E-15	1.00E-300	1.33E-94	6.91E-50	4.52E-215
GRU	XGBoost	1.64E-38	2.89E-46	6.80E-211	1.20E-62	1.00E-300
LSTM	SVR	6.92E-17	0.66079344	1.79E-97	2.26E-51	1.06E-274
LSTM	XGBoost	1.15E-40	1.00E-300	3.94E-213	5.29E-64	1.00E-300
MLP	GRU	2.89E-41	4.72E-12	1.63E-206	6.40E-75	1.00E-300
MLP	LSTM	1.85E-43	1.00E-300	1.00E-208	2.53E-76	1.00E-300
MLP	SVR	4.58E-35	1.00E-300	1.70E-85	3.76E-26	1.00E-300
MLP	XGBoost	0.07195239	2.70E-117	0.01888064	3.03E-07	6.20E-124
XGBoost	SVR	1.41E-32	1.00E-300	3.56E-86	3.34E-24	1.00E-300

Note: Author’s representation of the results.

**Table 14:** P values for the DM test on MAPE.

Model 1	Model 2	Onion	Paddy	Soybean	Tomato	Wheat
GRU	LSTM	2.58E-71	1.00E-300	3.40E-224	8.39E-45	1.00E-300
GRU	SVR	0.71049441	1.00E-300	1.22E-126	9.73E-07	1.19E-252
GRU	XGBoost	1.72E-101	8.40E-48	4.05E-269	2.10E-41	1.00E-300
LSTM	SVR	0.19659493	2.03E-06	2.31E-131	9.39E-08	1.00E-300
LSTM	XGBoost	3.37E-112	1.00E-300	2.64E-271	1.40E-45	1.00E-300
MLP	GRU	2.54E-126	1.70E-10	1.85E-263	2.54E-129	1.00E-300
MLP	LSTM	2.18E-135	1.00E-300	1.70E-265	4.04E-137	1.00E-300
MLP	SVR	5.17E-32	1.00E-300	3.86E-80	1.13E-14	1.00E-300
MLP	XGBoost	2.10E-05	1.49E-111	0.00731173	5.76E-09	3.12E-139
XGBoost	SVR	4.01E-33	1.00E-300	2.51E-80	3.40E-18	1.00E-300

The P-Values of the DM test, at a significance level of 5%, being less than 0.05 show that the comparisons of errors across models are statistically significant. The Diebold-Mariano (DM) test evaluates whether the predictive accuracy of two competing models differs significantly. The null hypothesis (​$\:{H}_{0})$ states that there is no significant difference in forecast errors between the two models, implying that they have equal predictive accuracy. The alternative hypothesis ($\:{H}_{A})$ suggests that one model has significantly lower forecast errors than the other. A low P-value indicates that the null hypothesis can be rejected, confirming that the difference in forecasting performance between the models is statistically significant.

### Discussion on accuracy results

Deep learning models, particularly LSTM and GRU, demonstrated outstanding accuracy across all error metrics, including Root Mean Squared Error (RMSE), Mean Absolute Error (MAE), and Mean Absolute Percentage Error (MAPE). These models are inherently designed to capture long-term dependencies and nonlinear patterns, which are prevalent in agricultural commodity prices due to factors like weather variability and market fluctuations. The LSTM model, for instance, consistently delivered lower RMSE and MAPE values, particularly for highly volatile commodities such as onions and tomatoes. GRU models, though slightly less robust than LSTM, also performed exceptionally well, with the added benefit of a simpler architecture that reduces computational overhead. The Echo State Network (ESN) emerged as another strong contender in the deep learning category. Unlike LSTM and GRU, ESN utilizes a fixed recurrent structure, training only the output layer. This approach not only improves computational efficiency but also provides strong performance in capturing dynamic temporal patterns. However, for certain commodities with highly complex price dynamics, ESN’s error metrics were marginally higher than those of LSTM and GRU, suggesting that while ESN is effective, it might require further tuning for optimal performance in such scenarios.

Traditional models like ARIMA and Linear Regression, while historically significant in time-series forecasting, exhibited clear limitations in this study. ARIMA, which relies on linear assumptions and stationarity, struggled to handle the nonlinear and non-stationary nature of agricultural price data. This limitation was reflected in its consistently higher RMSE and MAE values across most commodities. Linear Regression, a simplistic model that fits a straight line to data points, also failed to account for the inherent complexities and volatilities in the dataset, leading to suboptimal forecasting accuracy. Despite their limitations, these models occasionally delivered competitive results for commodities with relatively stable price patterns, such as paddy and wheat. This suggests that for less volatile commodities, the simplicity of ARIMA and Linear Regression may still provide acceptable performance, particularly when computational resources or model interpretability are prioritized.

Machine learning models like XGBoost and SVR occupied a middle ground in terms of performance. XGBoost, a powerful ensemble method based on gradient boosting, was particularly effective in handling moderate nonlinearities. Its performance across metrics like MAE and MAPE was commendable, especially for moderately volatile commodities such as coriander and soybean. However, XGBoost fell short of the deep learning models in capturing long-term temporal dependencies, which limited its effectiveness for highly volatile commodities. Support Vector Regression (SVR) also demonstrated decent performance, leveraging its kernel-based approach to model complex relationships within the data. However, its error metrics were consistently higher than those of LSTM and GRU, indicating a gap in capturing intricate patterns and dependencies. Additionally, SVR’s performance was sensitive to hyperparameter tuning, which could pose challenges in practical applications.

**Fig. 6:**
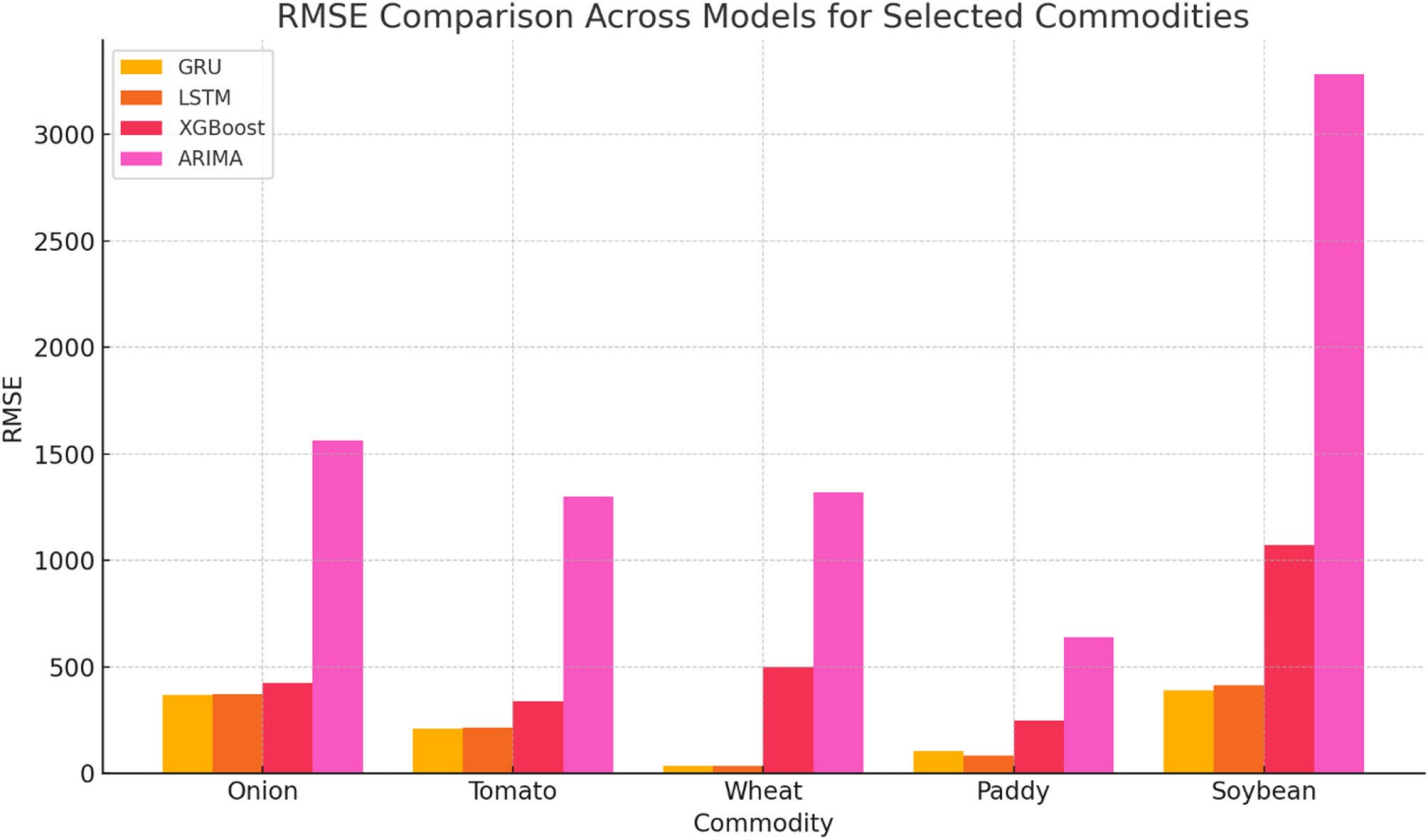
RMSE Comparison Across Models for Selected Commodities.

**Fig. 7:**
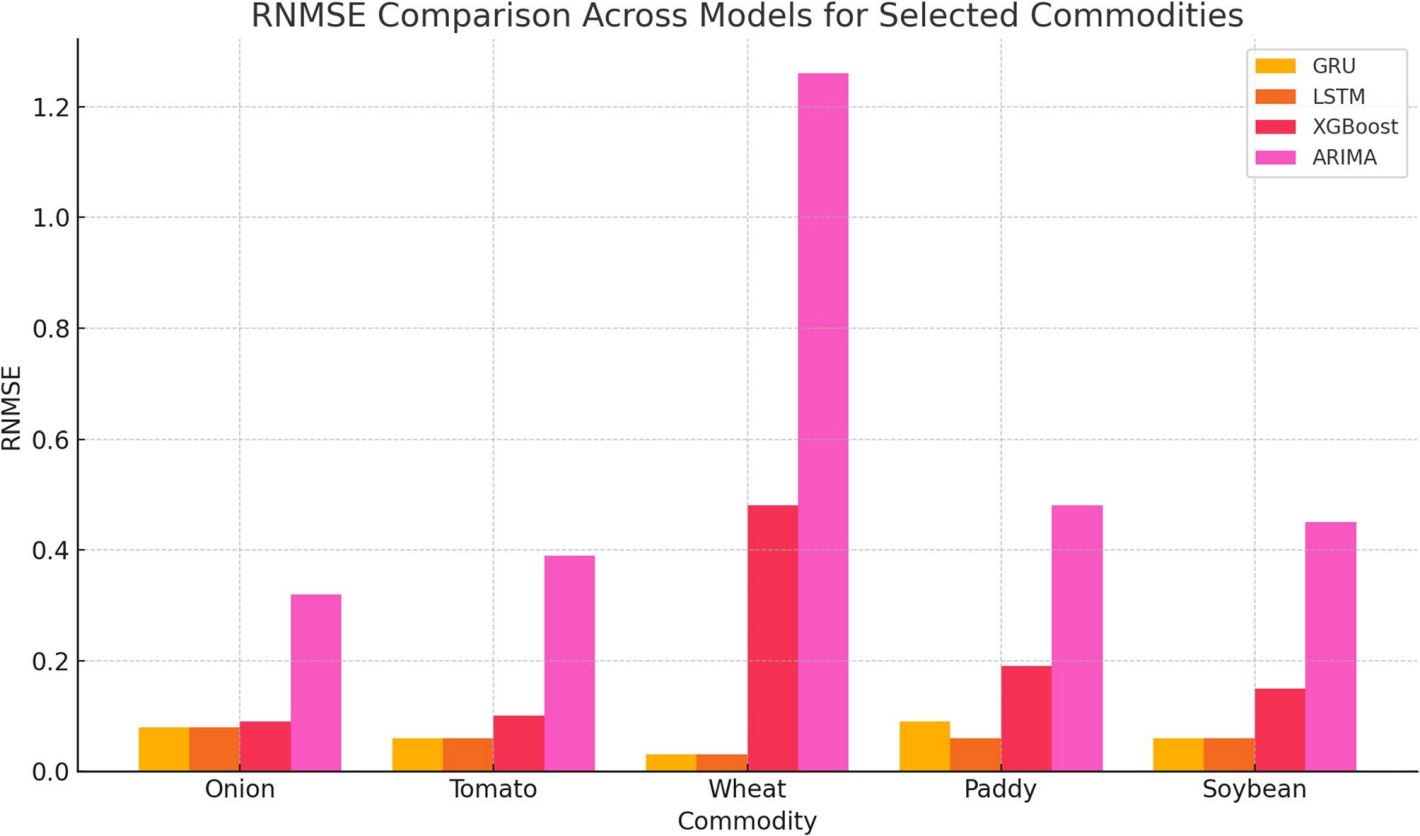
RNMSE Comparison Across Models for Selected Commodities.

**Fig. 8:**
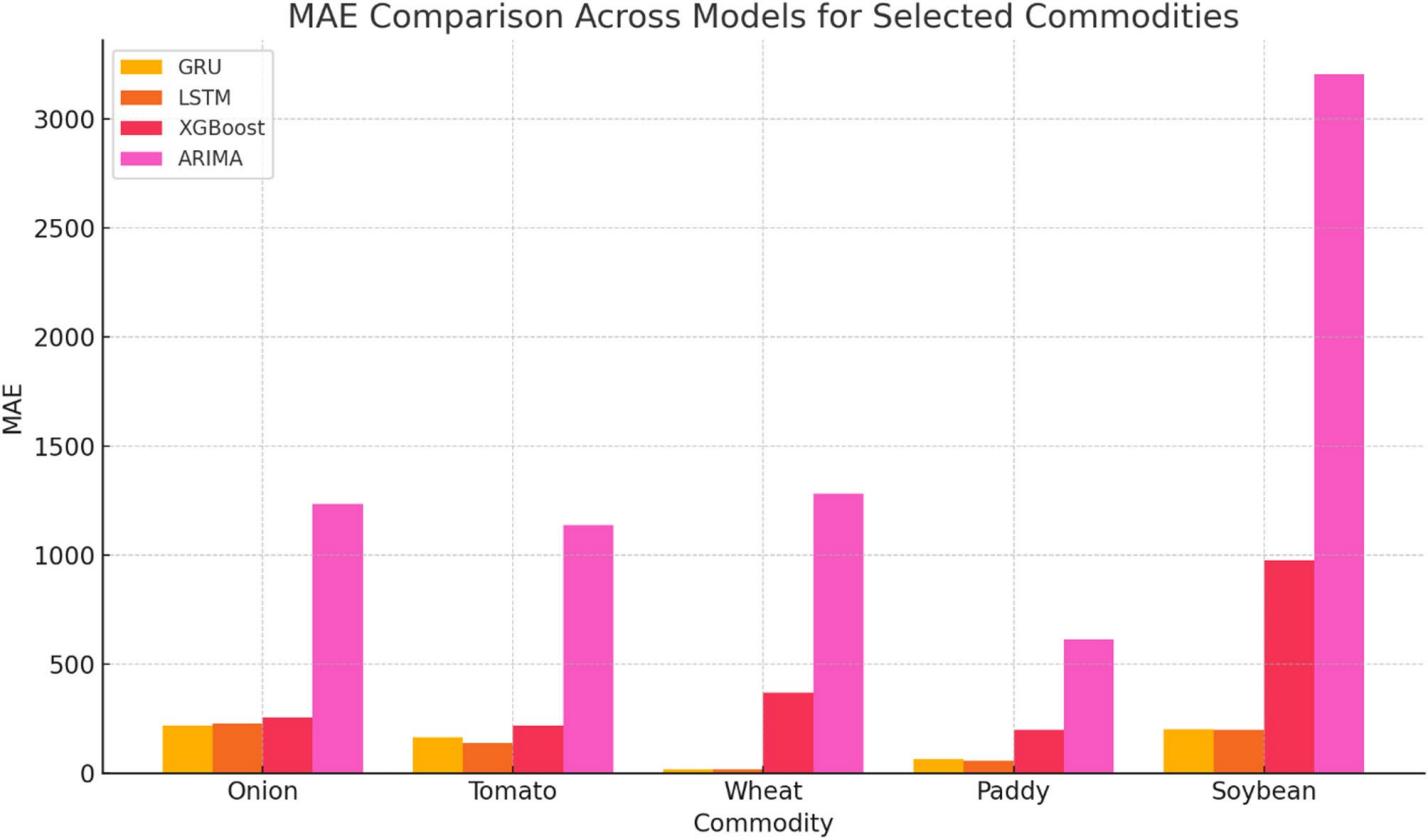
MAE Comparison Across Models for Selected Commodities.

**Fig. 9:**
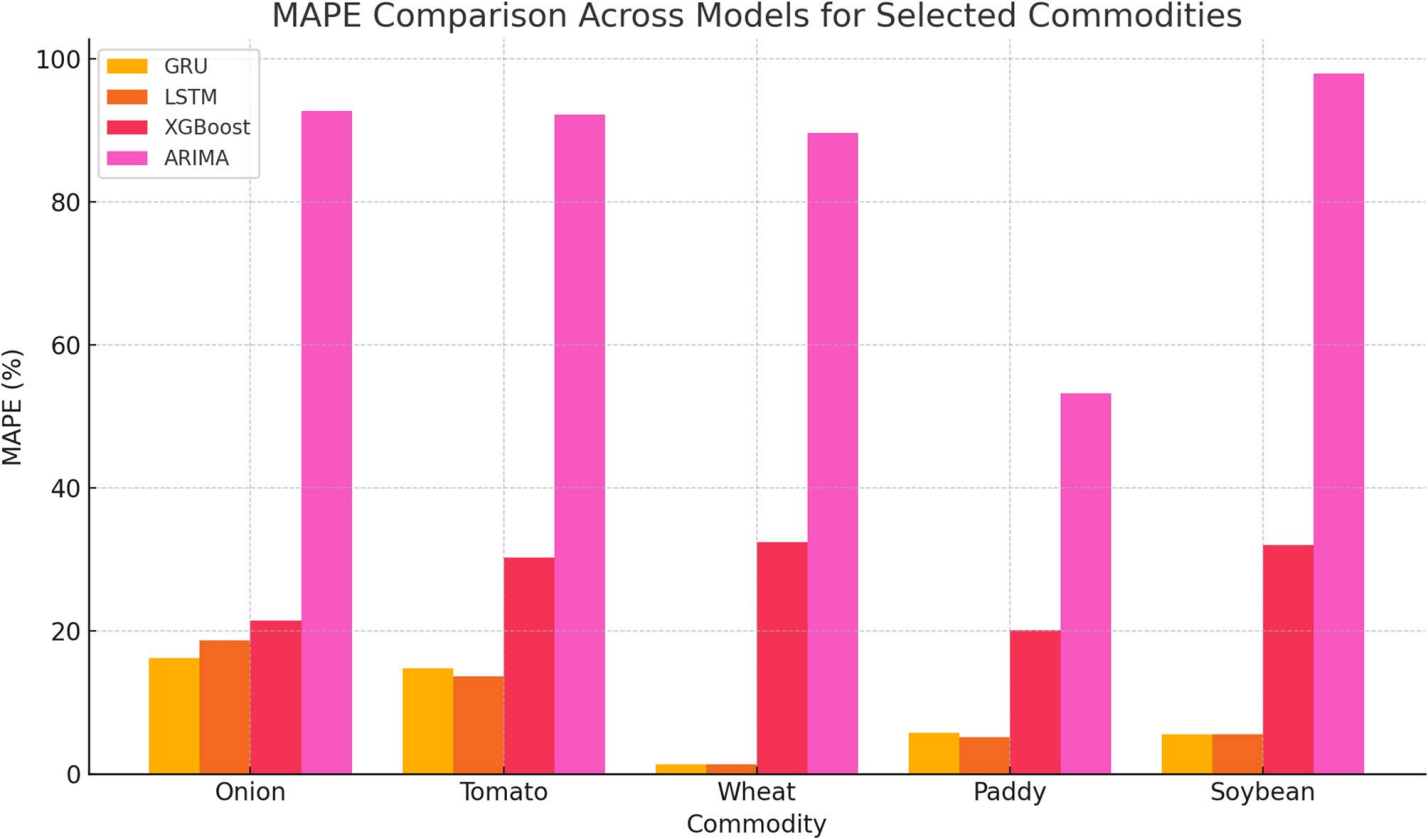
MAPE Comparison Across Models for Selected Commodities.

As shown in Fig. 6, GRU achieved the lowest RMSE values for onions (369.54) and tomatoes (210.35), significantly outperforming ARIMA, which recorded RMSE values of 1564.62 and 1298.60, respectively. Similarly, RNMSE comparisons in Fig. 7 reveal that GRU and LSTM models consistently maintained lower values across commodities, with GRU recording an RNMSE of 0.08 for onions and 0.06 for tomatoes, compared to ARIMA’s much higher RNMSE of 0.32 and 0.39 for the same commodities. These results underscore the ability of GRU and LSTM to handle price volatility effectively. MAE values, illustrated in Fig. 8, further emphasize the superiority of deep learning models. For instance, GRU and LSTM achieved MAE values of 217.73 and 229.70 for onions, respectively, while ARIMA recorded a much higher MAE of 1234.25. For tomatoes, GRU and LSTM showed MAE values of 164.28 and 139.30, respectively, compared to ARIMA’s 1138.77. This significant reduction in MAE highlights the precision of GRU and LSTM in forecasting commodity prices with minimal deviation from actual values. The MAPE analysis in Fig. 9 also provides critical insights into model performance. GRU recorded a MAPE of 16.17% for onions and 14.81% for tomatoes, significantly outperforming ARIMA’s MAPE values of 92.66% and 92.17%, respectively. These improvements are particularly important for highly volatile commodities, where accurate percentage error reductions can have substantial practical implications for stakeholders. The Echo State Network (ESN) also performed well, demonstrating competitive error metrics in capturing temporal patterns. However, as indicated in Figs. 6, 7, 8 and 9, its metrics were consistently marginally higher than those of GRU and LSTM, particularly for highly complex price dynamics, suggesting room for further optimization.

Traditional models like ARIMA and Linear Regression revealed significant limitations across all metrics, particularly for volatile commodities. While ARIMA achieved reasonable accuracy for relatively stable commodities like paddy and wheat, its RNMSE (0.48 for paddy) and MAPE (53.19% for paddy) remained considerably higher than those of GRU and LSTM. This limitation is evident across Figs. 7, 8 and 9, where ARIMA consistently lags behind deep learning models. XGBoost, as a machine learning model, occupied an intermediate performance level. While it performed better than ARIMA and Linear Regression in RNMSE, MAE, and MAPE, it could not match the performance of GRU and LSTM, particularly for volatile commodities. For instance, its RNMSE for onions was 0.09, slightly higher than GRU’s 0.08, as shown in Fig. 8.

The results highlight significant variations in model performance across different commodities. Highly volatile commodities like onions and tomatoes benefited the most from the advanced capabilities of LSTM and GRU models. These models effectively captured the sudden price spikes and drops, providing more accurate and reliable forecasts. For moderately volatile commodities such as wheat and soybean, machine learning models like XGBoost offered competitive performance, though deep learning models still held a slight edge. Interestingly, even for relatively stable commodities like paddy and maize, the advanced models outperformed traditional ones, underscoring the versatility and robustness of deep learning techniques. The superior performance of LSTM and GRU in these cases indicates their ability to generalize well across different volatility levels, making them suitable for a wide range of forecasting applications.

The superior performance of LSTM is due to its innovative memory cell architecture and gating mechanisms that allow it to capture both short-term fluctuations and long-term dependencies inherent in agricultural commodity price forecasting. LSTMs incorporate input, forget, and output gates that enable the network to selectively store and discard information, effectively mitigating the vanishing gradient problem and ensuring that crucial historical context—such as seasonal trends, policy shifts, and climatic events—remains influential over extended periods. This capability to model non-linear dynamics directly from raw data makes LSTM particularly adept at handling the complex interactions present in agricultural markets, where factors like weather variability and global economic conditions lead to unpredictable price movements. In contrast, while GRUs utilize a streamlined gating approach that offers computational efficiency, they may sometimes fall short in capturing very long-term dependencies due to their reduced complexity. Similarly, XGBoost, although powerful in handling structured data and complex feature interactions, relies heavily on manual feature engineering to incorporate temporal information and lacks an inherent mechanism to model sequential relationships. ARIMA, a classical statistical approach, is limited by its assumptions of linearity and stationarity, rendering it less effective in addressing the non-linear and volatile nature of agricultural price series.

The enhanced findings of this research have significant policy implications for agricultural market planning and regulation. Firstly, accurate price forecasting through advanced deep learning models like GRU and LSTM can empower policymakers to design more effective market stabilization strategies. Commodity price forecasting plays a crucial role in global markets, influencing trade policies, food security, and investment strategies^34^. By leveraging precise, timely predictions, authorities can implement dynamic pricing policies, such as minimum support prices (MSP) and targeted subsidies, ensuring that farmers receive fair compensation while minimizing the risk of market distortions. Historical examples in India underscore the critical need for reliable forecasting. For instance, during the 2013 pulses crisis, pulse prices in certain regions surged by as much as 200%, leading to an estimated loss in consumer surplus and market inefficiencies valued at roughly INR 3,500 crores. Similarly, the 2016 onion crisis saw prices in key producing states, such as Maharashtra, spike by nearly 300%, with market disruptions and losses estimated at around INR 12,000 crores. In both cases, the absence of robust forecasting tools contributed to reactive policy measures that amplified the economic fallout for both farmers and consumers.

For **farmers**, price forecasts can guide planting and marketing strategies. Research has shown that accurate forecasting models help stabilize agricultural markets and reduce risks for smallholder farmers^26^. A farmer armed with a forecast of low future prices might diversify crops or delay selling harvest (if storage is possible) to avoid losses. Conversely, a forecast of favorable prices could encourage planting more of that crop or selling forward to lock in profits. There are case studies where providing price outlooks to farmers improved their bargaining position – for example, some cooperative platforms share predictive insights, so farmers know if prices are likely to rise, helping them decide whether to bring produce to market immediately or hold off. Such decisions can materially affect farm income. Moreover, better forecasts reduce uncertainty, which can encourage investment in the agriculture sector. If both farmers and lenders trust price forecasts, credit and insurance products (like crop insurance or futures contracts) can be structured more effectively. In essence, forecast accuracy has an **economic multiplier effect**: it leads to more efficient market functioning. Stable and predictable prices allow for better planning of sowing area and crop rotation, optimal storage and logistics planning, and more stable consumer prices.

Smallholder farmers often lack access to sophisticated market information; improved forecasting (especially if disseminated via advisory services or apps) can level the playing field, enabling them to anticipate market gluts or scarcities. For example, a predictive model might warn of an expected bumper crop leading to price drops – farmers could then choose to process the crop or switch to an alternative to avoid a crash. On the policy side, accurate forecasts of cereal prices help governments ensure food **policy** decisions (like releasing public grain stocks or adjusting subsidies) are well-timed, preventing crises. A notable scenario is **food price inflation**: early prediction of a spike in a staple’s price lets policymakers act preemptively.

Furthermore, improved forecasting models enable policymakers to anticipate market volatility and supply chain disruptions, thereby enhancing food security planning. With better insights into potential price spikes or shortages, governments can proactively adjust import-export policies, manage strategic food reserves, and mobilize support for regions at risk of food insecurity. This proactive approach not only stabilizes markets but also protects consumers from sudden price hikes on essential commodities. Additionally, integrating these predictive analytics into national agricultural strategies can foster a more resilient and adaptive policy framework. By incorporating real-time data, including weather patterns and global market trends, into forecasting systems, governments can better coordinate multi-sectoral responses that mitigate the impact of both climatic and economic shocks. This comprehensive, data-driven approach will support sustainable agricultural growth, enhance market transparency, and ultimately contribute to long-term economic stability and food security.

## Conclusion

This study utilized three categories of models to predict the prices of various commodities: stochastic, machine learning, and deep learning. The stochastic models, such as ARIMA, are limited to short-term memory and cannot adequately capture dataset volatility. In contrast, the data-driven machine learning and deep learning models’ approaches are capable of handling the data’s nonlinearity and complexity. The 23 commodities analyzed exhibit unique price behavior patterns. The exploration of these techniques showed that deep learning methods consistently outperformed both machine learning and stochastic models across all patterns. This superiority is attributed to deep learning’s ability to capture both long-term and short-term memory patterns in the data, as well as nonlinear complexities.

The findings of this research also have significant policy implications for agricultural market planning and regulation. Accurate price forecasting can help policymakers design more effective interventions to stabilize markets and support farmers. For instance, timely and reliable price predictions enable the implementation of minimum support prices (MSP) and other subsidies that ensure farmers receive fair compensation for their produce, reducing their vulnerability to market volatility. Moreover, enhanced forecasting accuracy can improve food security planning by helping authorities anticipate and mitigate the effects of price spikes and shortages on consumer access to essential commodities. Policies that leverage advanced predictive models, such as GRU and LSTM networks, can be more proactive and adaptive, providing better tools for managing supply chain disruptions and enhancing overall market resilience.

Despite the promising results, this study has limitations that warrant further investigation. Firstly, the predictive models used rely heavily on historical price data, which may not fully capture sudden, unforeseen market shocks caused by extreme weather events, geopolitical tensions, or pandemics. Incorporating real-time data and integrating exogenous variables—such as weather forecasts, global market trends, policy changes, and socio-political disruptions—into the forecasting models could significantly enhance their robustness and adaptability to dynamic market conditions. Additionally, while deep learning models like GRU and LSTM have shown superior performance, their computational requirements and data preprocessing demands may pose challenges for certain stakeholders, particularly in resource-constrained settings or regions with limited access to advanced computing infrastructure.

Future research will explore the development of hybrid models that combine the strengths of traditional statistical methods with advanced machine learning and deep learning techniques. Hybrid approaches could leverage the interpretability and simplicity of stochastic models while integrating the nonlinear learning capabilities of deep learning methods. Moreover, these models could incorporate diverse data types, including both quantified variables (e.g., historical prices, weather data, and market indices) and non-quantified inputs (e.g., text data from market reports, news sentiment analysis, and policy announcements). Such enhancements could improve prediction reliability, computational efficiency, and scalability for a wider range of users.

Expanding the scope of analysis to include a broader set of agricultural commodities, regional market data, and cross-border trading patterns would also provide deeper insights into global and regional agricultural dynamics. This broader scope could enable more comprehensive models that account for interdependencies across markets and commodities. Furthermore, collaborative efforts to make advanced forecasting tools more accessible to policymakers, small-scale farmers, and other stakeholders through user-friendly interfaces and educational outreach could maximize the practical utility of these models. By addressing these gaps, future studies can pave the way for more resilient, adaptive, and equitable agricultural markets.

## Data Availability

The datasets used and/or analysed during the current study available from the corresponding author on reasonable request.
